# Co-adjuvant nano particles for hepatocellular carcinoma radiotherapy treatment

**DOI:** 10.1186/s43046-025-00300-3

**Published:** 2025-06-14

**Authors:** Ramy Sabry Abd-Elsamee, Doaa Ezzat Sayed Ahmed, Khalid Shaaban Hashem, Ahmed Nabil

**Affiliations:** 1 Biotechnology and Life Sciences Department, Faculty of Postgraduate Studies for Advanced Sciences (PSAS), Beni-Suef University, Beni-Suef, Egypt; 2 College of Oral and Dental Surgery, Misr University for Science & Technology, Beni-suef, Egypt; 3 Department of Biochemistry, Faculty of Veterinary Medicine, Beni-Suef University, Beni-Suef, Egypt

**Keywords:** Selenium nanoparticles, Hepatocellular carcinoma, Ultra violet, Reactive oxygen species, Transmision electron microscopy, Scanning electron microscopy, Dimethylthiazole assay

## Abstract

HCC is one of the most life-threatening human cancers in the world. It is considered the major malignant tumor of the liver in adults and is the most common cause of death in people with cirrhosis. Chemotherapy is widely used for HCC treatment, but it has many side effects. Therefore, an alternative, safe method with low side effects, low toxicity, and a higher anti-cancer effect is in demand. In our study, we used Se-NPS alone and combined it with Gamma and UV radiation at different doses. We also used the chemotherapeutic drug sorafenib on Hep G2 cell lines to compare the effect of Se-NPS (with and without radiation) with the sorafenib group. Our results showed that Selenium alone without radiation had a lesser effect on eliminating cancer cells, with high cell viability and fewer apoptotic effects. On the other hand, Selenium combined with radiation, especially at high doses of UV (180 s) and gamma (0.2 Gy), had the highest effect on killing cancer cells. This combination resulted in significantly lower cell viability, high DNA fragmentation, and a high apoptotic effect due to a significant elevation of P53 and cytosolic cytochrome C, which was better than the Radiation-only groups.

## Introduction

The primary liver cancer, HCC ranks fourth globally in terms of cancer-related deaths and is the primary cause of cancer-related deaths in low-resource nations [1]. Globally, hepatocellular carcinoma incidence rates vary, with over 80% of all cases occurring in low- and middle-resource nations. East Asia and sub-Saharan Africa have very high incidence rates [2]. Before plateauing in recent years, the disease’s age-adjusted incidence rates in the USA tripled between 1992 and 2010 [3, 4]. The high frequency of chronic hepatitis C virus infection in the population cohort born between 1945 and 1965 and the significantly elevated burden of metabolic syndrome are probably the causes of the increased incidence rates [5].

The US Food and Drug Administration has approved sorafenib (SFB), the first molecularly targeted medication, for the initial therapy of advanced, incurable HCC [6]. In order to achieve an effective anti-tumor effect, it works to inhibit angiogenesis, cancer cell proliferation, and the activity of several tyrosine kinase receptors, such as fibroblast growth factor receptor (FGFR), platelet-derived growth factor receptor (PDGFR), and vascular endothelial growth factor receptor (VEGFR) [7]. Like many targeted medications, SFB has intrinsic drawbacks, such as low solubility in water and vulnerability to the body’s metabolic breakdown. As a result, higher dosages of SFB are needed in clinical settings to guarantee its effectiveness against HCC [8]. However, clinical research has shown that long-term SFB use can result in a number of negative consequences, such as rash, diarrhea, high blood pressure, and other harmful side effects [9]. As a result, a new therapeutic approach is desperately needed.

The use of nanomaterials in nanobiotechnology is well known for its several significant applications, particularly in drug delivery and diagnostic systems [7], as well as the prosthetics and implants. Since most biological systems are also nanoscale, it is possible to integrate nanoscale material types with biomedical equipment in an effective manner. Carbon nanotubes, metal and inorganic nanoparticles, metallic surfaces, and liposomes are the materials typically used in the creation of those nanotechnology goods [8].

It is possible to conjugate bio-specific molecules with nanoparticles by using physical or chemical methods and taking advantage of certain biological events, such as antibody–antigen contacts, DNA–DNA hybridization, and receptor–ligand interactions.

For inoperable tumors, radiotherapy (RT) based on very penetrating MeV photons (X-rays and g-rays) is beneficial and non-invasive. Since HCC is a tumor that is sensitive to radiation, treatment plays a crucial part in the thorough therapy of HCC [9]. However, the tumor selectivity of radiation is weak. All tissues are susceptible to photon damage, which can have detrimental consequences on the healthy liver tissue that surrounds the tumor. These patients are more vulnerable to lower radiation doses than those with normal livers because they frequently have a history of cirrhosis. Radiation-induced liver disease (RILD) is a major risk to patients'lives, and the incidence of radiotherapy problems increases as radiation dose increases [10]. Therefore, the goal of future cancer radiotherapy is to simultaneously improve the radiation's bioavailability and the tumor tissues’ selectivity. By increasing the sensitivity of tumor cells to radiation, radiosensitizers can build up in the tumor tissue, increasing the likelihood that tumor cells will be destroyed by lower radiation doses [11]. The creation of nanoparticles (NPs) is a crucial step in the development of numerous medications that have been produced as HCC radiosensitizers [12]. Local therapy of solid tumors can result from the use of NPs that exhibit preferential aggregation in tumors (even when passively absorbed because of enhanced permeability and retention effects, or EPR) [13]. Additionally, it has been suggested that NPs with a high Z atom are promising radiosensitizers because, when combined with various radiation types of varying intensities, they may have potent radiosensitizing effects on tumors [14]. Furthermore, after a century of research on the biological foundation of RT, five key factors—namely, (1) the ability of cells to repair cellular damage, (2) the ability of cells to repopulate, (3) the redistribution of cell cycles, (4) cell reoxygenation, and (5) radiosensitivity—were implicated in defining the overall effect of RT on tumors [15]. According to published research, high-Z metallic nanoparticles (NPs) like selenium (Se), gold (Au), bismuth (Bi), and gadolinium (Gd) have the capacity to induce radiosensitization [16].

Due to the inclusion of selenoproteins, selenium (Se) is a trace element that is vital for numerous cellular processes [17]. When compared to organic and inorganic selenium, selenium nanoparticles (SeNPs) are becoming more and more significant due to their lower toxicity, bioavailability, protein interaction, and biocompatibility [18]. At large dosages, they are renowned for their strong anticancer properties [19].

Due to its exceptional antioxidant capacity, superior biocompatibility, reduced toxicity, and cancer prevention effects, selenium nanoparticles (SeNPs) have garnered a lot of attention recently as a possible anti-tumor agent and drug carrier [20, 21]. Se exhibits unique pyroelectricity, piezoelectricity, non-linear optical sensitivity, and photoconductivity as a trace element [22]. Se’s function in chemotherapy has been thoroughly investigated.

SeNPs have a wide range of anti-cancer properties and can concurrently increase the formation of ROS [23].In particular, SeNPs reinforced radiosensitivity, G2/M phase cell cycle arrest, and autophagy activation [24]. SeNPs, a novel radiosensitizer, increased the killing impact of tumor cells and decreased normal tissue damage when paired with irradiation treatment. The combination of 6 Gy radiation treatment with SeNPs (3 μg/ml) boosted apoptosis by 14.94% and cell death by 29.67% compared to either SeNPs or 6 Gy radiation alone [25]. According to recent studies, selenium (Se) has the potential to lessen the negative side effects of radiation without sacrificing the efficacy of treatments [26]. The stimulation of various ROS-mediated signaling pathways was also discovered to be a mechanism for selenium compounds to efficiently increase the anticancer efficacy of ultraviolet radiation [27, 28]. Based on SeNPs’ intriguing physical and biochemical properties as well as their therapeutic benefits, we postulated that exposure to UV light would increase ROS production and provide synergistic anticancer effects.

By applying SeNPs alone and in combination with varying UV and gamma radiation dosages to Hep G2 cell lines and comparing the results with those of sorafenib medication and untreated Hep G2 cell lines, we hope to demonstrate the efficacy of SeNPs as a potent radio sensitizer in the treatment of HCC.

## Materials and methods

### Nano selenium preparation

Selenium nanoparticles were synthesized via chemical reduction using sodium selenite as a precursor and ascorbic acid as a reducing agent. The reaction was conducted in aqueous medium at pH 7–8 under constant stirring, where the gradual color change to brick-red indicated nanoparticle formation. After completion, the SeNPs were collected by high-speed centrifugation, washed with distilled water and ethanol, and redispersed in deionized water using ultrasonication. Particle characteristics were controlled by adjusting parameters such as pH, reactant concentration, and temperature [29].

### Se NPs nanothymol physicochemical characterization

#### Transmisionelectron microscopy (TEM)

By creating a standardized spontaneous emulsion solution of thymol nanoparticles for sonication in a 250-ml glass beaker (height 9.5 cm, diameter 7 cm), nanothymol (2% w/v), saponin as solvent, and distilled water, the physicochemical features of the thymol nano-emulsion were investigated. A 100 ml reaction solution was made while being constantly stirred. The MCS Digital Ultrasonic Cleaner 2500 ml, with heater, was used to standardize the ultrasonic treatment parameters for this thymol nanoemulsion. During sonication at a pulse rate (on/off) of 5 s, the probe was submerged in the center of a glass beaker and allowed to descend 1.75 cm at a regulated temperature below 35 °C. The reaction liquid turned milky white and a translucent dispersion emerged after 8–10 min of sonication, demonstrating the homogeneity and stability of the thymol nanoemulsion at room temperature for up to 50 min. To prevent the sedimentation and agglomeration of thymol nanoparticles, a dispersion agent was added [30, 31].

To make the suspensions, 0.01–1 mg of anothymol were dissolved in 1 ml of Tween as solvent, and then distilled water was added until the volume reached one liter. In short, ethanol (2% w/w), ethyl acetate (9% w/w), tween-80 (27% w/w), thymol (2% w/w), and ultrapure water (60 w/w) were added while being continuously stirred. The TEM technique was used to physically characterize thymol nanoparticles. To prevent coarse particles, the suspension was filtered through the proper filters. In order to maximize concentration and prevent excessive agglomeration, droplets of the aqueous nanosuspension were subsequently micropipetted onto a Lactic carbon-coated copper grid (Lacy for 3D imaging) (Electron Microscopy Sciences) on a filter paper support [32, 33]. To reduce nanoparticle movement and aggregation, the solvent was allowed to gently evaporate under carefully regulated circumstances (such as room temperature and low humidity). These operations were carried out at Mansoura University’s Faculty of Agriculture’s Electron Microscopy Unit in Mansoura, Egypt.

#### Scanning electron microscopy

SEM is a type of electron microscope in which a high-energy electron beam is scanned in a raster to create an image on a substance. The signals generated when the electrons make contact with the constituent atoms of the sample reveal its surface topography, composition, electrical conductivity, and other properties. By stabilizing with BSA and reducing with sodium selenite glutathione (reduced form), SeNPs were produced. The nanoparticles (Se) were sterilized by UV radiation in a laminar flow. The sterile nanoparticles were carefully positioned on SEM stubs using adhesive tape. Nanoparticle samples were placed in the JEOL JSM-6490 LV sample chamber of the SEM and scanned at 15,000–35,000 × and voltages ranging from 20 to 30 kV [34, 35]. These procedures were conducted at the Faculty of Agriculture at Mansoura University. Electron Microscopy Unit in Mansoura, Egypt.

#### Zeta potential

Zeta potential was analyzed for SeNPs at the Unit of Electron Microscopy, Faculty of Agriculture, Mansoura University, Mansoura, Egypt.

### Cell lines preparations

At 37 °C in an incubator with 5% CO2, the HepG2 cell line was cultivated in RPMI-1640 media supplemented with 10% fetal bovine serum (FBS), 100 units/mL penicillin, and 50 units/mL streptomycin. The gamma radiation was 0.1 Gy (Gray) and 0.2 Gy, the UV radiation was 90 and 180 s, and the dose of selenium nanoparticles was 750 μg/ml.

#### Experimental design

HepG2 cell lines used as a model for HCC in the form of untreated Hep G2 (control group),DMSO group, and 10 groups of samples prepared.

Control group Hep G2 cell lines, DMSO group Hep G2 cell lines with DMSO, Group 1 HepG2 cell lines with sorafenib drug only. Group 2 HepG2 cell lines with selenium nanoparticles and without radiation. Group 3 HepG2 cell lines with Gamma 0.1 radiation and without selenium nanoparticles. Group 4 HepG2 cell lines with Gamma 0.2 radiation and without selenium nanoparticles. Group 5 HepG2 cell lines with UV 90 s radiation and without selenium nanoparticles. Group 6 HepG2 cell lines with UV 180 s radiation and without selenium nanoparticles. Group 7 Hep G2 cell lines with selenium nanoparticles and Gamma radiation 0.1. Group 8 Hep G2 cell lines with selenium nanoparticles and Gamma radiation 0.2. Group 9 HepG2 cell lines with selenium nanoparticles and UV 90 s radiation and Group 10 Hep G2 cell lines with selenium nanoparticles and UV 180 s radiation. Each experiment has been replicated 3 times.

#### Cell viability by MTT assay

MTT dissolves in buffered salt solutions, ethanol (20 mg/ml), water (10 mg/ml), and medium (5 mg/ml). When kept at 0 °C, reconstituted MTT solutions can be kept for at least 6 months. More than 4 days of storage at 4 °C will induce deterioration and produce inaccurate results.

96-well test: 96-wellassay: at least three times, repeat each condition.

Day 1: Trypsinize one flask and fill it with 5 ml of full medium. Centrifuge 15 ml sterile Falcon tubes for 5 min at 2000 rpm. Add 250 µl of 5000 cells to each well, remove the medium, suspend the cells in 1.0 ml of complete medium, count and record the number of cells per ml, and then incubate for the entire night.

Day 2: remove the medium, rinse with PBS, and provide the medication of interest to the cells in accordance with the previously mentioned experimental design. Day 3: empty the medium, rinse with PBS, and fill each well with 25 μl of 5 mg/ml MTT. At 37 °C, incubate for 2 h in a CO2 incubator. Measure the absorbance at 590 nm using a reference filter at 620 nm after adding 50 μl of DMSO and incubating for 15 min at 37 °C CO2 [36].

Viability of cells (%) = (meanOD-controlOD) × 100.

### Cell cycle analysis by flowcytometry

#### Preparation of tissue suspension for cell cycle analysis and P^53^ detection

Fresh tissue samples were prepared in accordance with [36], and they were brought to our lab in isotonic saline. The specimens were rinsed in a 0.1-M Tris (hydroxyl methylaminomethane) solution containing 3.029 g, 1.022 g of 0.07 M sodium chloride (ADWIC), and 0.005MEDTA 0.47 g. After dissolving them in 250 ml of distilled water, 1 N HCl was used to bring the PH down to 7.5. After centrifuging the cell suspension for 10 min at 1800 rpm, the supernatant was aspirated. If there was macroscopic blood, it was lysed for 10 min using filtered tap water. Following centrifugation and aspiration of the supernatant, the cells are preserved for approximately 1 ml of each sample in ice-cold 96–100% ethanol (BDH).

#### Staining procedures

Collect cells, suspend them in PBS, add cold ethanol drop by drop until the final concentration is 70%, fix them on ice for at least 2 h, wash them in PBS, suspend them in staining buffer (PBS plus 100 μg/mL RNase A, 50 μg/mL propidium iodide, and optionally 0.1% Triton X-100), wrap them in foil and shield them from light, incubate them overnight at 4 °C, and record the results on a flow cytometer (Becton Dickinson, Sunnyvale, CA, USA).

### P^53^ analysis by flowcytometry

#### Staining procedures

Gather cells, suspend them in PBS, add cold ethanol drop by drop until the final concentration is 70%, fix them on ice for at least 2 h, wash them in PBS, suspend them in staining buffer (PBS plus 100 μg/mL RNase A, 50 μg/mL propidium iodide, and optionally 0.1% Triton X-100), add 2 to 10 μl of anti-P53 Abs conjugated to fluorescein isothiocyante (FITC), wrap them in foil and shield them from the light, incubate them overnight at 4 °C, and record the results on a flow cytometer (Becton Dickinson, Sunnyvale, CA, USA).

### DNA fragmentation

#### DNA extraction

DNA was extracted from tissue homogenates (200 mg) using the Zymo Research Quick-g DNATM Mini Prep kit (Catalog No. D3024). The tissue homogenate was centrifuged for 10 min at 4 °C at 12,000×*g*. DNA isolation was performed using the leftover supernatant. To create Genomic Lysis Buffer, 50 ml of buffer was mixed with 250 µl of β-mercaptoethanol. The tissue homogenate supernatant was mixed with 400 µl of Genomic Lysis Buffer. To fully mix, these were vortexed for four to 6 s. For 5 to 10 min, the mixture was left to stand at room temperature. Two hundred microliters of DNA Pre-Wash Buffer was added to the spin column and centrifuged at 10,000×*g* for 1 min after the mixture was moved to a Zymo-SpinTM Column in a Collection Tube and the Collection Tube was disposed of with the flow-through. The spin column was filled with 500 µl of DNA Wash Buffer and centrifuged for 1 min at 10,000×*g*. Sixty microliters of DNA Elution Buffer was added to the spin column after it had been moved to a sterile microcentrifuge tube. Following 2 to 5 min of incubation at room temperature, DNA was extracted using centrifugation for 30 s at maximum speed. For later use, eluted DNA was kept at − 20 °C. The optical density (OD) of the isolated DNA was measured with a spectrophotometer set to 260 nm UV.

#### Detection of the extracted DNA using agarose gel electrophoresis

Agarose gel electrophoresis was used to identify the DNA products at the conclusion of the extraction procedure. Biometra hybrid horizontal gel tank. ultraviolet light source. Gel recording system. Biometra’s Biodocand Analyser. The following is how 2% agarose gels were made: in a flask covered with aluminum foil, 1 g of agarose was dissolved in 50 ml of TAE buffer and cooked for 5 min at a medium temperature in a microwave oven. Gloves were used when handling solutions containing EB dye because it is a potent mutagen and mildly poisonous. 2.5 μl of EB was added to the agarose solution after it had cooled to 60 °C in order to see DNA. Clean, dry gel molds were filled with gels. The gel was put in the electrophoresis chamber after the comb was gently removed after the agarose had thoroughly rested for 20 to 30 min. To allow the DNA to flow through the gel toward the positive electrode, the sample wells were positioned at the negative electrode of the gel chambers. Enough TAE buffer was then injected to cover the gel. The electrophoresis chamber's lid was shut, and the gels were run for 20 min at 100 V and 250 amps. A 100 by DNA ladder (Jena Bioscience, Germany) was used to mount gels on a UV transilluminator, and the DNA recorded by the gel recording device was visualized.

### Cytochrome C detection by Western blotting

Cytochrome C detected in each group in mitochondria and cytoplasm according to the following methods.

#### Separation of mitochondria from tissue cell lines

Transfer the cell pellet to a 15-mL Dounce homogenizer after suspending it in 11 mL of ice-cold RSB hypobuffer. Alternately, as explained in [37], use a Teflon pestle to transfer 3 mL of cells at a time to a 5-mL Potter-Elvehjem homogenizer after suspending the cell pellet in 9 mL of ice-cold RSB hypo-buffer. Give the cells 5 to 10 min to swell. Examine the swelling’s development with a phase-contrast microscope. After many B pestle strokes, swollen cells will shatter. Press the pestle directly onto the tube with each stroke, keeping the pressure firm and steady.

If a Potter-Elvehjem homogenizer was used in Step 1, a Teflon pestle spinning at 1600 rpm breaks the cells. Use a phase-contrast microscope to examine the degree of homogenization. A few intact cells (big spheres that seem granular), organelles (dark granular objects), and naked nuclei (smooth spheres with evident nucleoli inside) should all be present if cell lysis was successful. A very good result is eight to nine bare nuclei per entire cell. The number of mitochondria caught in the nuclear pellet during the initial centrifugation and the number of damaged nuclei will often grow with any greater outcomes. With a final concentration of 1 × MS homogenization buffer, 8 mL of 2.5 × MS homogenization buffer should be added right away. Parafilm should be placed over the top of the homogenizer and invert it many times to combine. In a gentle manner, centrifuge the homogenate after transferring it to a centrifuge tube. Add the homogenizer to the homogenate after rinsing it with a small amount of 1×MS homogenization buffer. Use 1×MS homogenization buffer to get the volume to 30 mL. To get rid of big membrane pieces, whole cells, and nuclei, centrifuge the homogenate for 5 min at 1300×*g*. Fill a sterile centrifuge tube with the supernatant. After transferring the supernatant to a sterile centrifuge tube, pellet the mitochondria for 15 min at 7000–17,000 g. Throw away the supernatant and use a Kimwipe to clean the tube’s interior. Suspend the mitochondrial particles in 1 × MS buffer and repeatedly sediment at 7000–17,000 g to clean the mitochondria. To prepare the pellet for further work, resuspend it in a buffer and discard the supernatant. For at least a year, mitochondria can be kept at – 80 °C, depending on the use (e.g., protein isolation).

#### Separation of cytoplasm from tissue cell lines

Every preparation was done on ice. After being suspended in 1 milliliter of hypotonic solution with 0.1% NP-40, the cells were incubated for 3 min. After being suspended in a solution, the cells were incubated for 3 min. The cells were then homogenized by repeating the pestle up and down 20 times using a Potter-Elvehjem homogenizer. To create pellet the nuclei, the fluid was centrifuged for 5 min at 1000 cf. To pellet the debris, the supernatant (cytoplasmic fraction) was centrifuged again at 15,000 rcf for 3 min. A hypotonic solution was added to the cells for 3 min in order to undertake fractionation with non-ionic detergent. After that, digitonin or NP-40 was added to reach a final concentration of 0.1%. After 3 min, the resultant solution was centrifuged at 1000 rcf for 5 min. To precipitate debris, the cytoplasmic fraction (supernatant) was centrifuged again at 15,000 rcf for 3 min.

#### Protein extraction

Following the manufacturer’s instructions, the ReadyPrep TM Protein Extraction Kit (total protein) from Bio-Rad Inc. (Catalog No. 163–2086) was added to each sample of homogenized tissue from every group. Bio Basic Inc. (Markham, Ontario, L3R 8 T4 Canada) supplied the Bradford Protein Assay Kit (SK3041) for quantitative protein analysis. To ascertain the protein concentration in each sample, the Bradford assay was carried out in accordance with the manufacturing instructions. Each sample’s 20 μg protein content was placed into an equal volume of two Laemmli sample buffers, which contained 0.125 M TrisHCl, 4% SDS, 10% 2-mercaptoethanol, 20% glycerol, and 0.004% bromophenol blue. After checking, the pH was set to 6.8. To ensure protein denaturation, each of the aforementioned mixtures was boiled for 5 min at 95 °C before to loading onto polyacrylamide gel electrophoresis.

#### Cytochrome C detection

Bio-Rad Laboratories Inc.’s TGX Stain-FreeTM FastCastTM Acrylamide Kit (SDS-PAGE), Cat # 161–0181, was used to perform polyacrylamide gels. The SDS-PAGE The preparation of TGX Stain-Free FastCast was done in accordance with the production guidelines; the same was true for TGX Stain-Free FastCast for SDS-PAGE. Filter paper, PVDF membrane, gel, and gel were combined in transfers and sandwiches from bottom to top. The sandwiches were then put in a transfer tank with one transfer solution, which contained 25 mM Tris, 190 mM glycine, and 20% methanol. The protein bands from the gel were then transferred to the membrane by blotting at 25 V for 7 min using a BioRadTrans-Blot Turbo. Membranes were blocked for 1 h at room temperature in Tris-buffered saline containing 3% bovine serum albumin (BSA) and Tween 20 (TBST) buffer. The following were the ingredients of the blocking buffer: 3% bovine serum albumin (BSA), 150 mM NaCl, 0.1% Tween 20, and 20 mM Tris pH 7.5. We bought cytochrome C primary antibodies. The manufacturer’s recommendations were followed while diluting primary antibodies in TBST.3–5 times, blots were rinsed in TBST for 5 min. The blotted target proteins were incubated for 1 h at room temperature in a solution of goat anti-rabbit IgG-HRP-1 mg Goat mab (Novus Biologicals) conjugated with HRP.3–5 times, blots were rinsed for 5 min with TBST.

Following the manufacturer’s instructions, a chemiluminescent substrate (Clarity TM Western ECL substrate Bio-Rad cat#170–5060) was applied to the blots. In short, a CCD camera-based imager was used to record the chemiluminescence signals after equal volumes of solutions A (Clarity Western luminal/enhancer solution) and B (peroxidase solution) were introduced. Using image analysis software, protein normalization was carried out on the ChemiDoc MP imager in order to read the target protein's band intensity in relation to the housekeeping protein, β-actin, in the control sample.

## Results

### Se-nanoparticles characterization results

The characterization of nano-thymol by TEM (JEOL-JEM 2100) by dipping a lacey carbon coated copper grid into the sample powder, revealed its spherical morphology with minimal or no aggregation, for total 18 particles from this field.

**Fig. 1:**
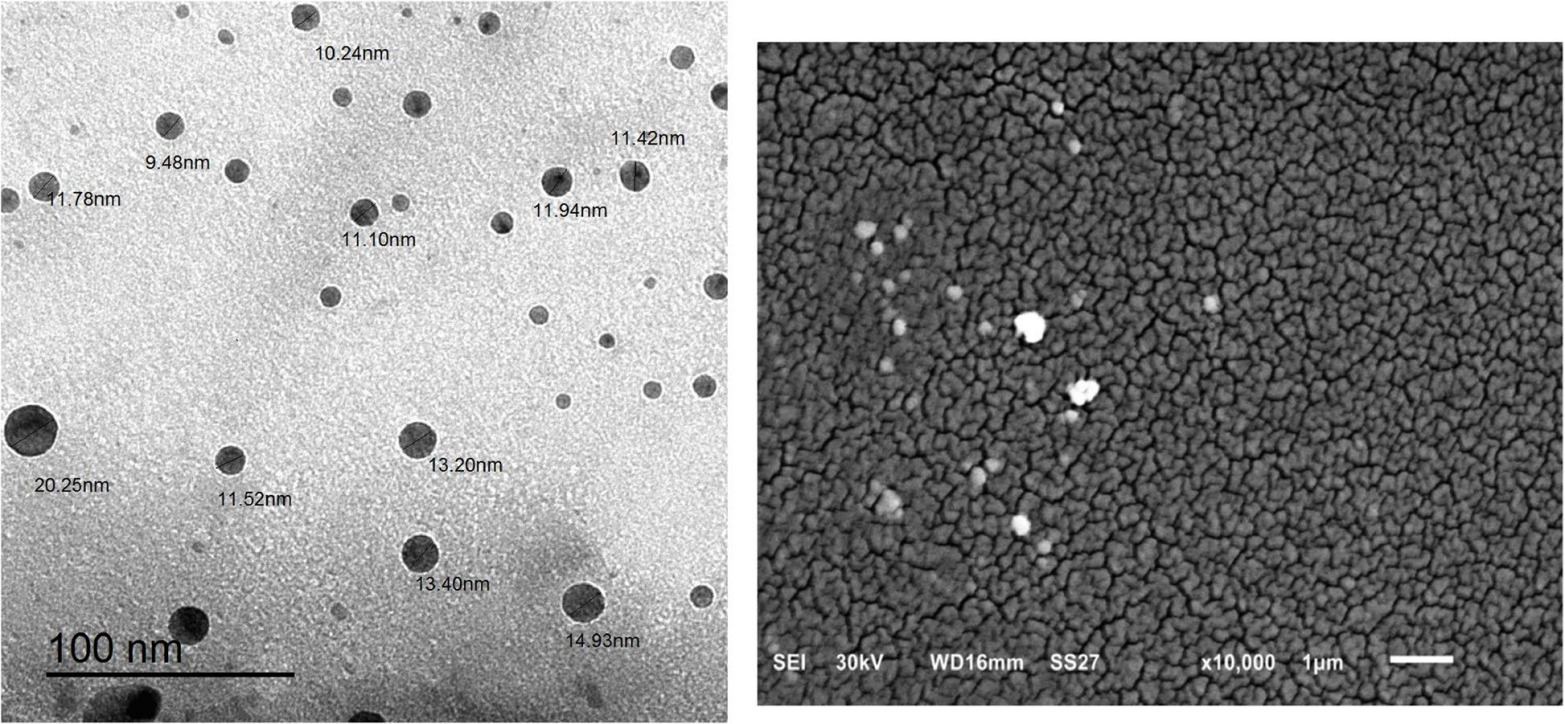
TEM for Se NPTs showing size on the left and SEM for Se NPTs on the right showing spherical and un aggregated shape of Se NPTs

**Fig. 2:**
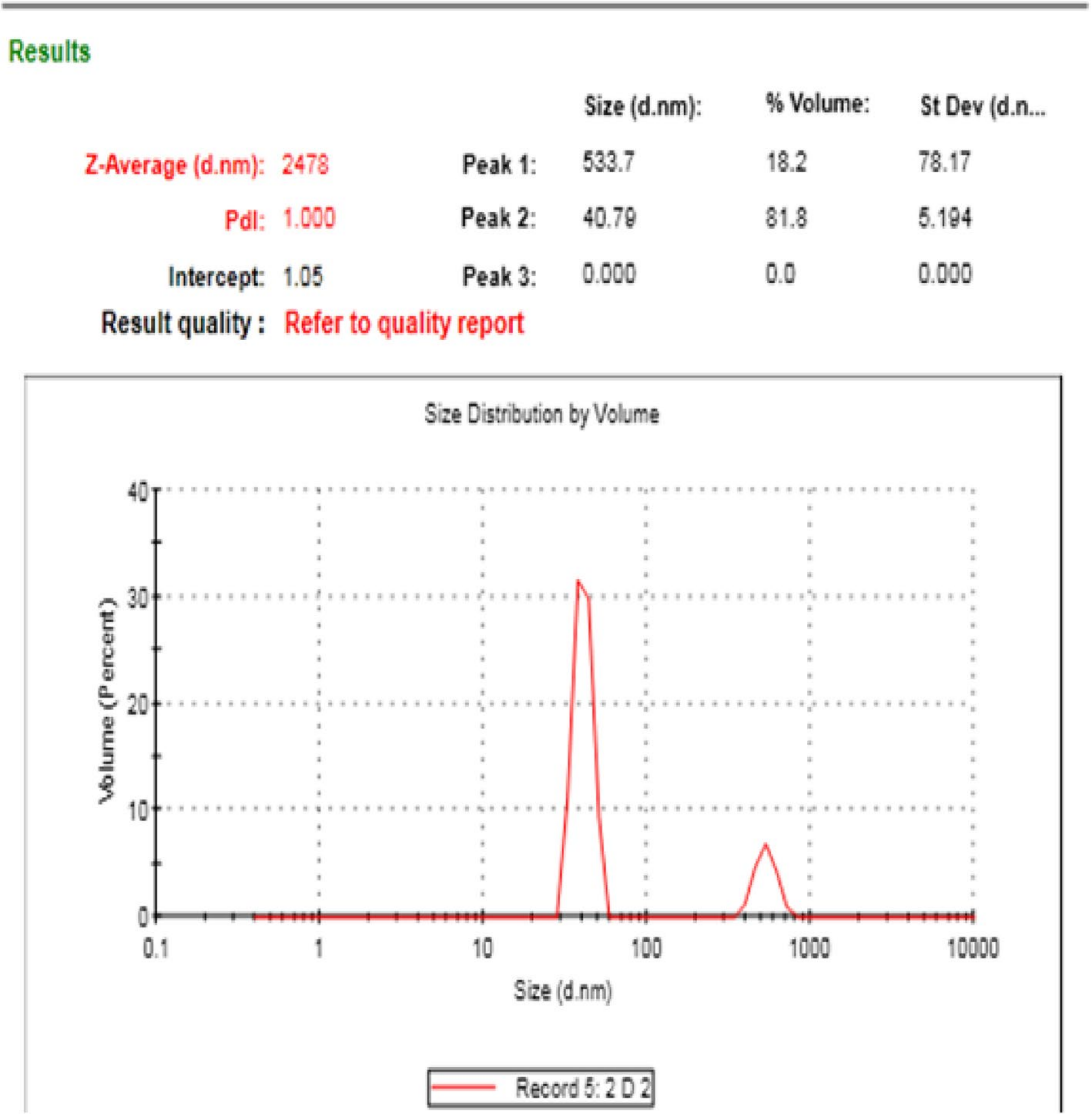
The average Zeta size distribution

**Fig. 3:**
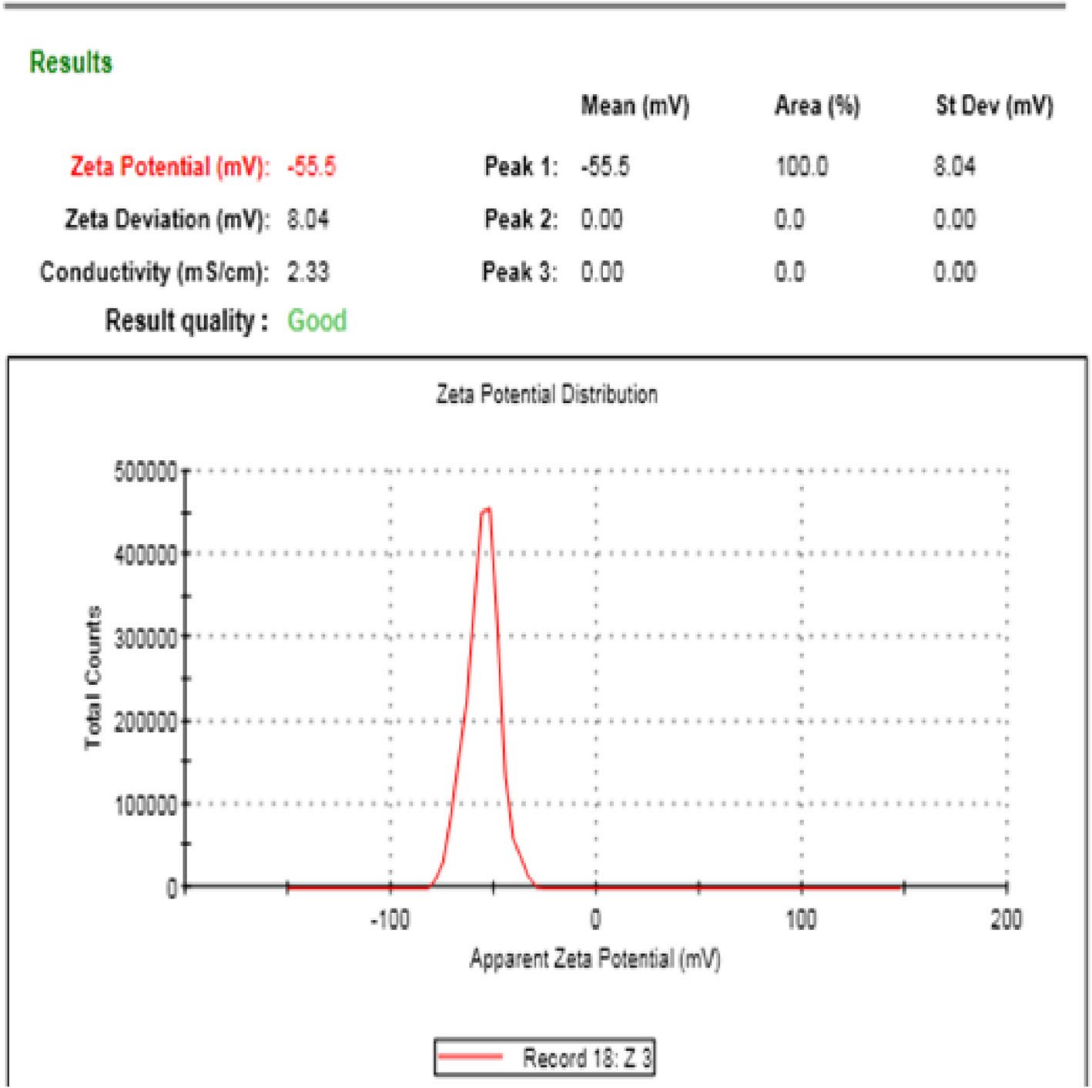
The average Zeta potential distribution

The mean particle size is 12.66 nm (Fig. 1). The average Zeta size distribution of nano-thymol by *Zetasizer Nano S*, Malvern, presented with mean value 651.8 in diameter (Fig. 2). PDI is 1.07. The mean of zeta potential was − 6.39 mV. The mean of zeta deviation was 7.35 mV as shown in Fig. 3. These measurements were carried out at the Unit of Electron Microscopy, Faculty of Agriculture, Mansoura University, Mansoura, Egypt.

### MTT results

Group 1 has a significant result compared to control group. Group 2 has a significant result compared to control group and group 1. Group 3 has a significant result compared to control, group 1, and group 2. Group 4 has a significant result compared to control, DMSO, group 1, group 2, and group 3. Group 5 has significant results compared to control, group 1, and group 4. Group 6 has a significant result compared to control, group 2, group 4, and group 5. Group 7 has significant results compared to control, group 1, group 2, and group 4. Group 8 has significant results compared to control, group 1, group 2, and group 4. Group 9 has significant results compared to control, group 1, group 4, and group 6. Group 10 has significant results compared to control, group 1, group 2, group 4, group 5, group 7, group 8, and group 9 (*P*-value (ANOVA) < 0.0001).

**Fig. 4:**
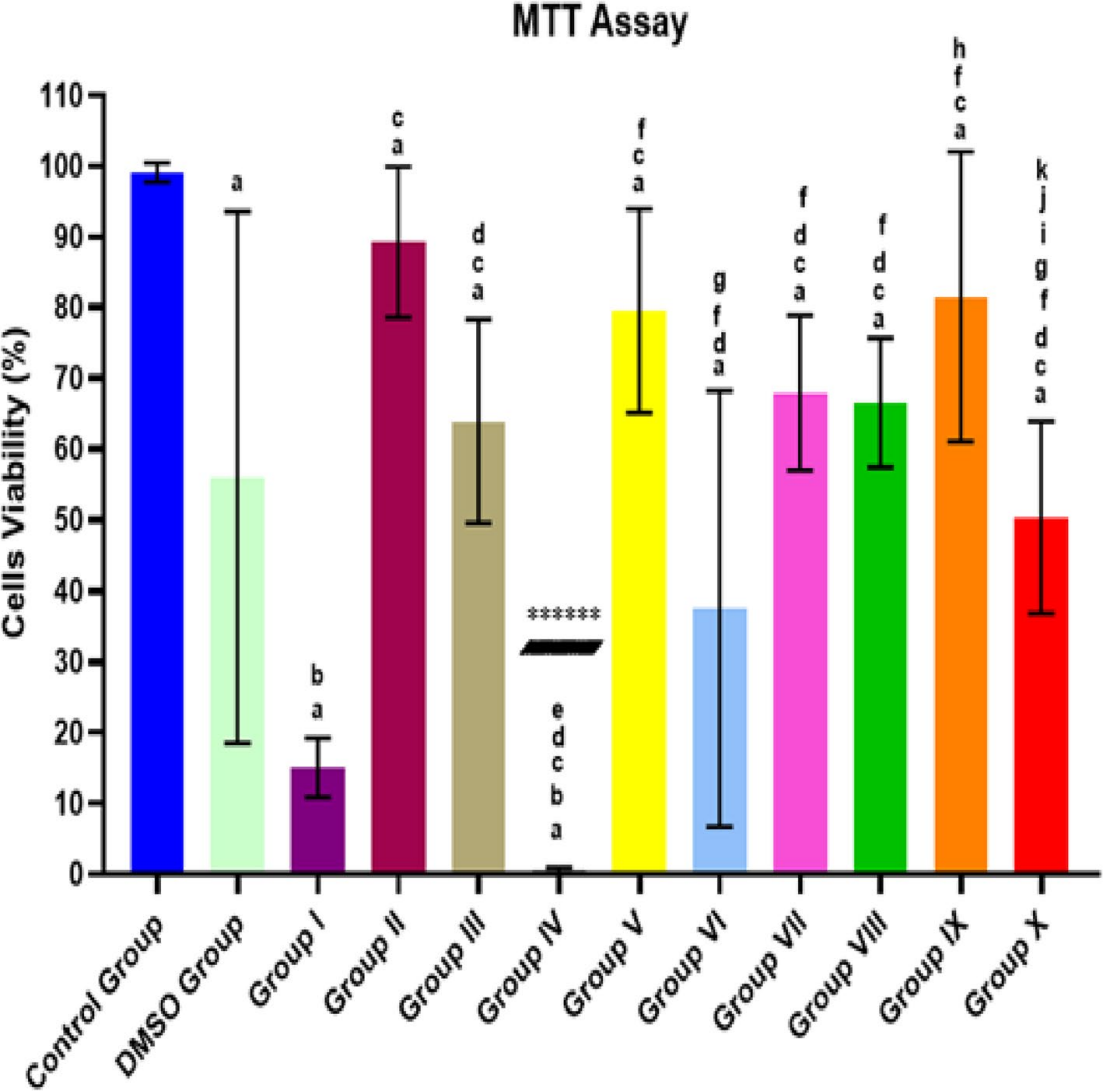
MTT assay results

According to Fig. 4 and Table 1, the Sorafenib group’s cell viability was lower than that of the Selenium groups with radiation, selenium groups, and radiation groups, with the exception of group 4 (Hep g2 with gamma radiation 0.2). Selenium mixed with radiation groups, particularly group 4 (gamma 0.2) and group 6 (UV 180), demonstrated greater cell viability than radiation groups lacking selenium in groups 3, 4, 5, and 6. When combined with UV and gamma radiation, selenium nanoparticles reduce the viability of cancer cells while also lessening the strong effects of radiation on cancer cells. This makes selenium nanoparticles an excellent choice for protecting healthy cells from harm in close proximity to cancer cells.

**Table 1:** MTT assay precise values

Groups	MTTassay (cells viability %)
Control group	99.1 ± 1.4
DMSO group	56.1 ± 37.5^a^
Group I (Sor)	15.1 ± 4.18^ab^
Group II (SeNPs)	89.2 ± 10.7^ac^
Group III (Gamma 0.1)	63.9 ± 14.4^acd^
Group IV (Gamma 0.2)	0.58 ± 0.36^abcde^
Group V (UV 90)	79.5 ± 14.4^acf^
Group VI (UV 180)	37.5 ± 30.8^adfg^
Group VII (SeNPs+Gamma 0.1)	67.9 ± 10.9^acdf^
Group VIII (SeNPs+Gamma 0.2)	66.5 ± 9.1^acdf^
Group IX (SeNPs+UV 90)	81.5 ± 20.5^acfh^
Group X (SeNPs+UV 180)	50.3 ± 13.6^acdfgijk^
*P*-value (ANOVA)	< 0.0001

^a^Significant with control group

^b^Significant with DMSO group

^c^Significant with group I

^d^Significant with group II

^e^Significant with group III

^f^Significant with group IV

^g^Significant with group V

^h^Significant with group VI

^i^Significant with group VII

^j^Significant with group VIII

^k^Significant with group IX

### P53 and cell cycle analysis by flowcytometry

#### P53 analysis

**Fig. 5:**
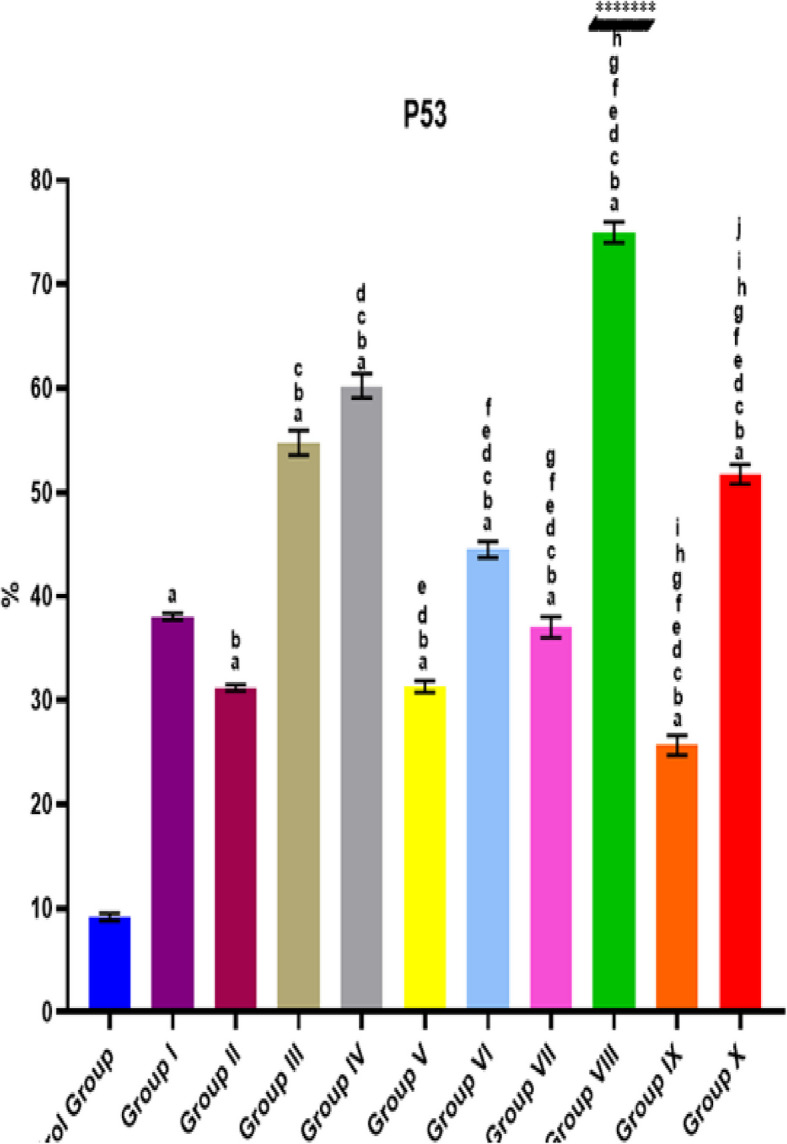
P53 results

**Fig. 6:**
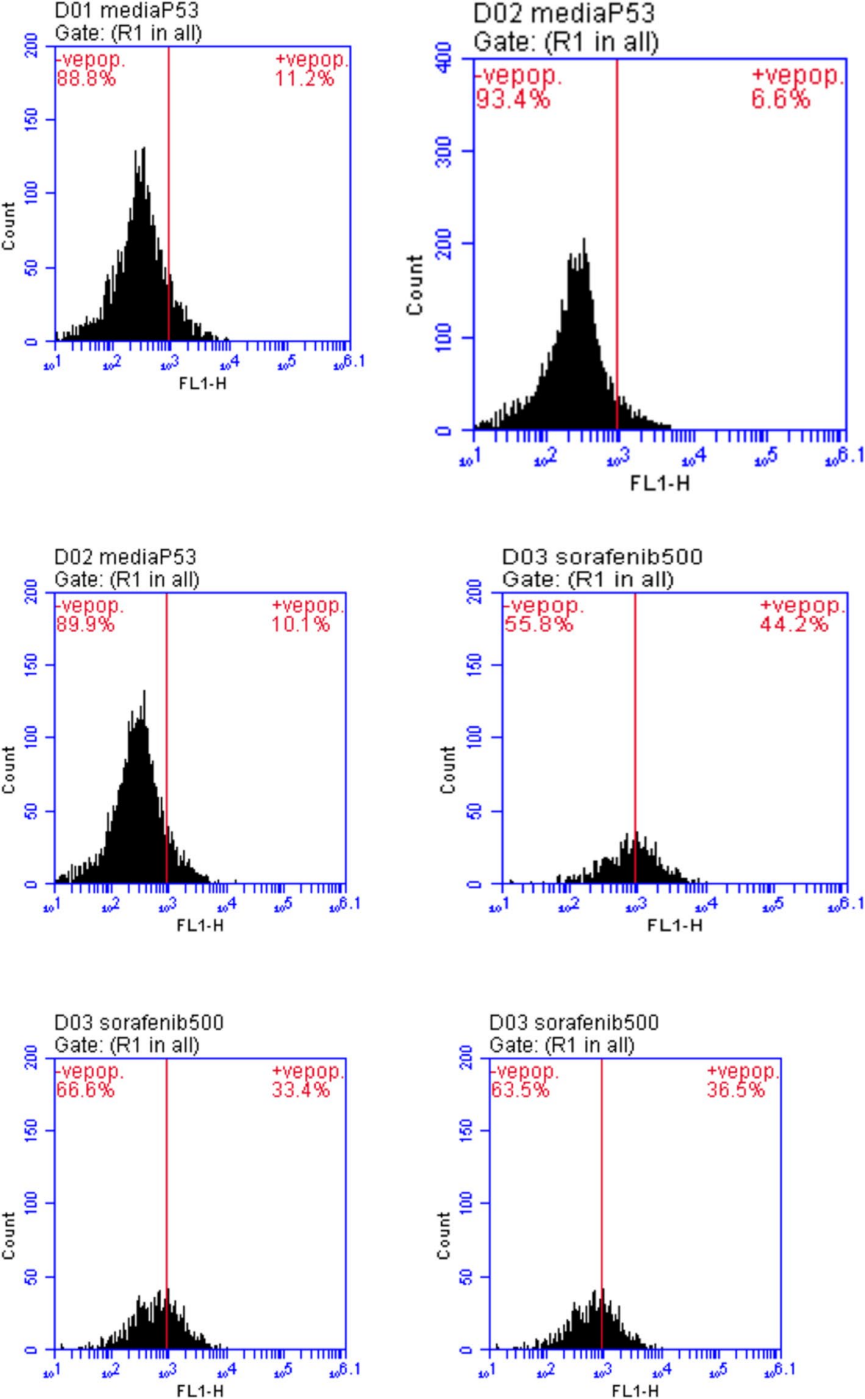
Flowcytometric histogram blot for P53 percent in control group and sorafenib group

**Fig. 7:**
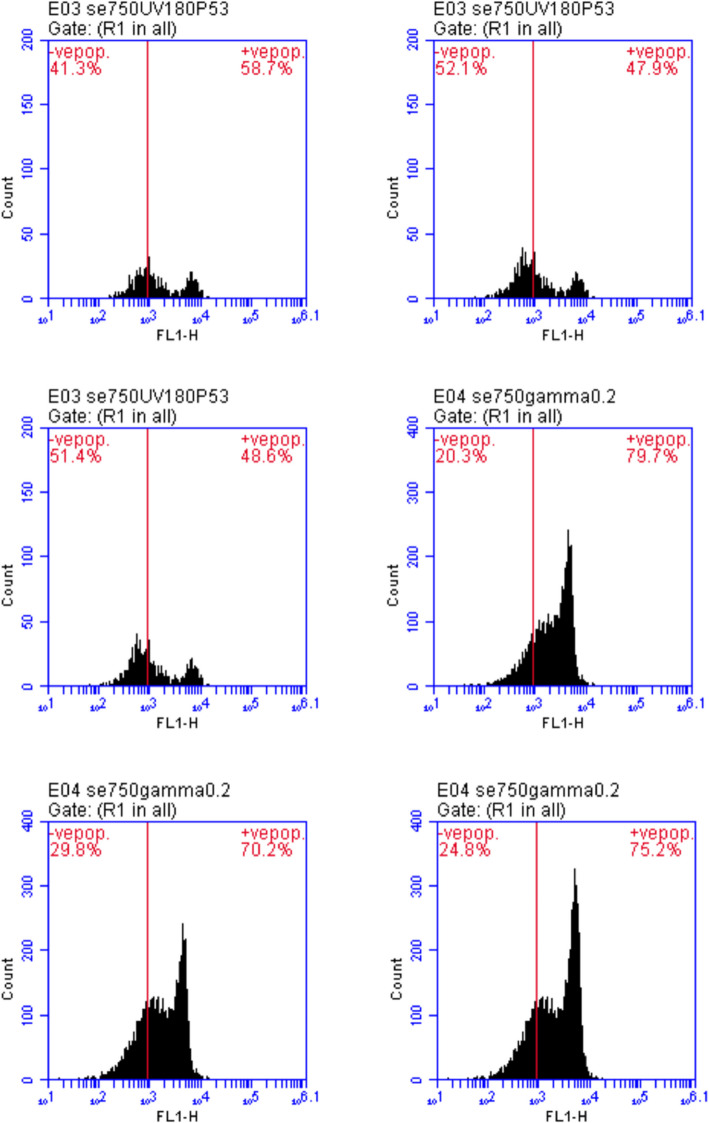
Flowcytometric histogram blot for P53 percent in Se 750 UV180 and Se 750 gamma 0.2 group

**Fig. 8:**
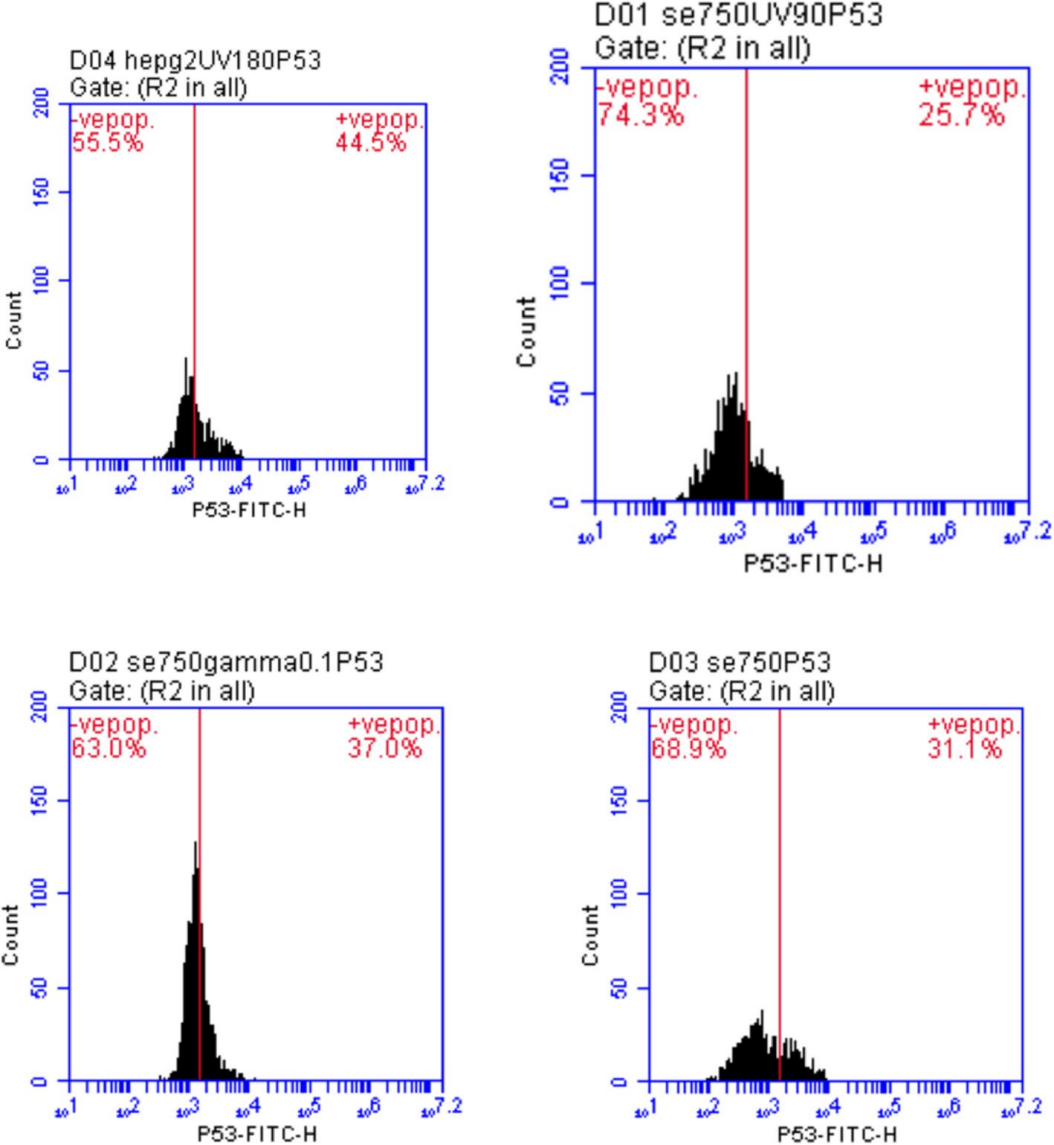
Flowcytometric histogram blot for P53 percent in Se 750, Se750 UV90, and Se 750 gamma 0.1 group

**Fig. 9:**
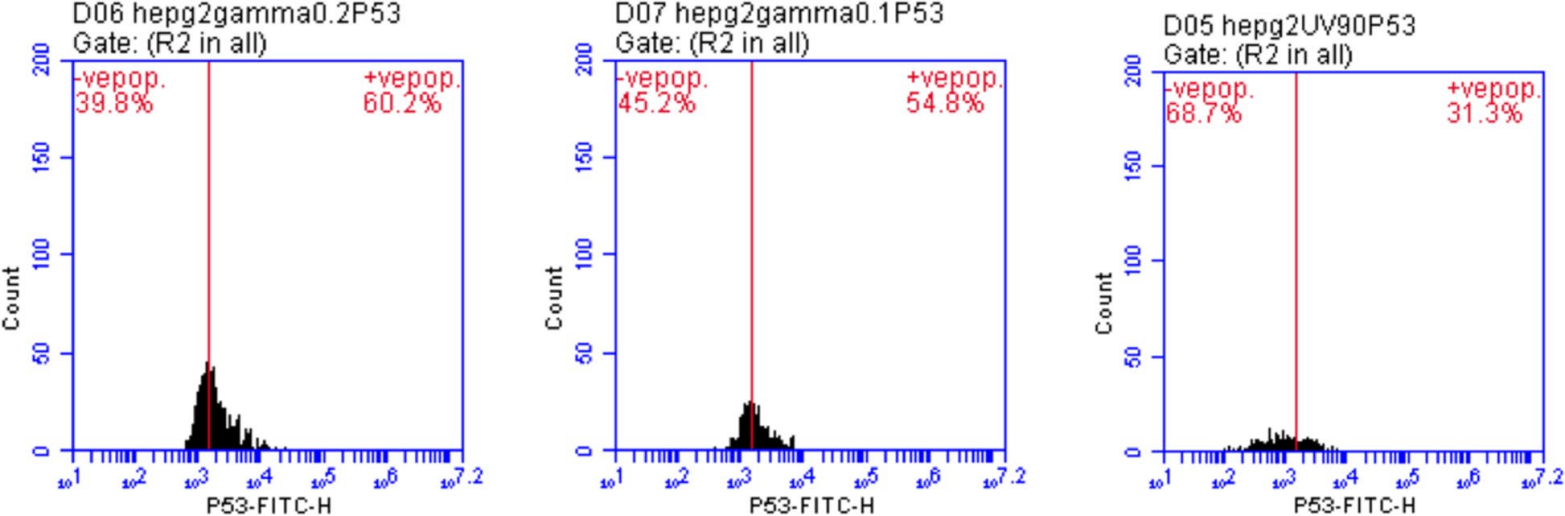
Flowcytometric histogram blot for P53 percent in Hep g2 UV180, Hep g2 gamma 0.1, and Hep g2 gamma 0.2 group

As shown in Fig. 5, according to flowcytometric histogram blots in (Figs. 6, 7, 8, and 9), the higher concentration of p53 found in groups 8, 4, 3, 10, 6, 1, 7, 2, 5, 9, and control group respectively. There is a significant difference between control group and experimental groups there is a significant difference between group 1.

According to Fig. 5 and Table 2, the concentration of P53 in the control group (Hep G2 only) was significantly lower than that of the other groups, while the highest concentration of P53 was found in group 8 (HepG2 Se gamma 0.2), which was significantly higher than the sorafenib group. Thus, the greatest tumor suppression and apoptosis that took place in group 8 (Se gamma 0.2).

**Table 2:** P53 precise values

Groups	P53 (%)
Control group	9.17 ± 0.35
Group I (Sor)	38 ± 0.32^a^
Group II (SeNPs)	31.2 ± 0.3^ab^
Group III (Gamma 0.1)	54.8 ± 1.2^abc^
Group IV(Gamma 0.2)	60.2 ± 1.7^abcd^
Group V(UV 90)	31.3 ± 0.6^abde^
Group VI(UV 180)	44.5 ± 0.78^abcdef^
Group VII(SeNPs+Gamma 0.1)	37 ± 1^abcdefg^
Group VIII(SeNPs+Gamma 0.2)	75 ± 1^abcdefgh^
Group IX(SeNPs+UV 90)	25.7 ± 0.97^abcdefghi^
Group X(SeNPs+UV 180)	51.7 ± 0.95^abcdefghij^
*P*-value (ANOVA)	< 0.0001

Sorafenib group and groups 3, 4, 6, 8, and 10 (*P*-value (ANOVA) < 0.0001

^a^Significant with control group

^b^Significant with DMSO group

^c^Significant with group I

^d^Significant with group II

^e^Significant with group III

^f^Significant with group IV

^g^Significant with group V

^h^Significant with group VI

^i^Significant with group VII

^ j^Significant with group VIII

### Cell cycle analysis

**Fig. 10:**
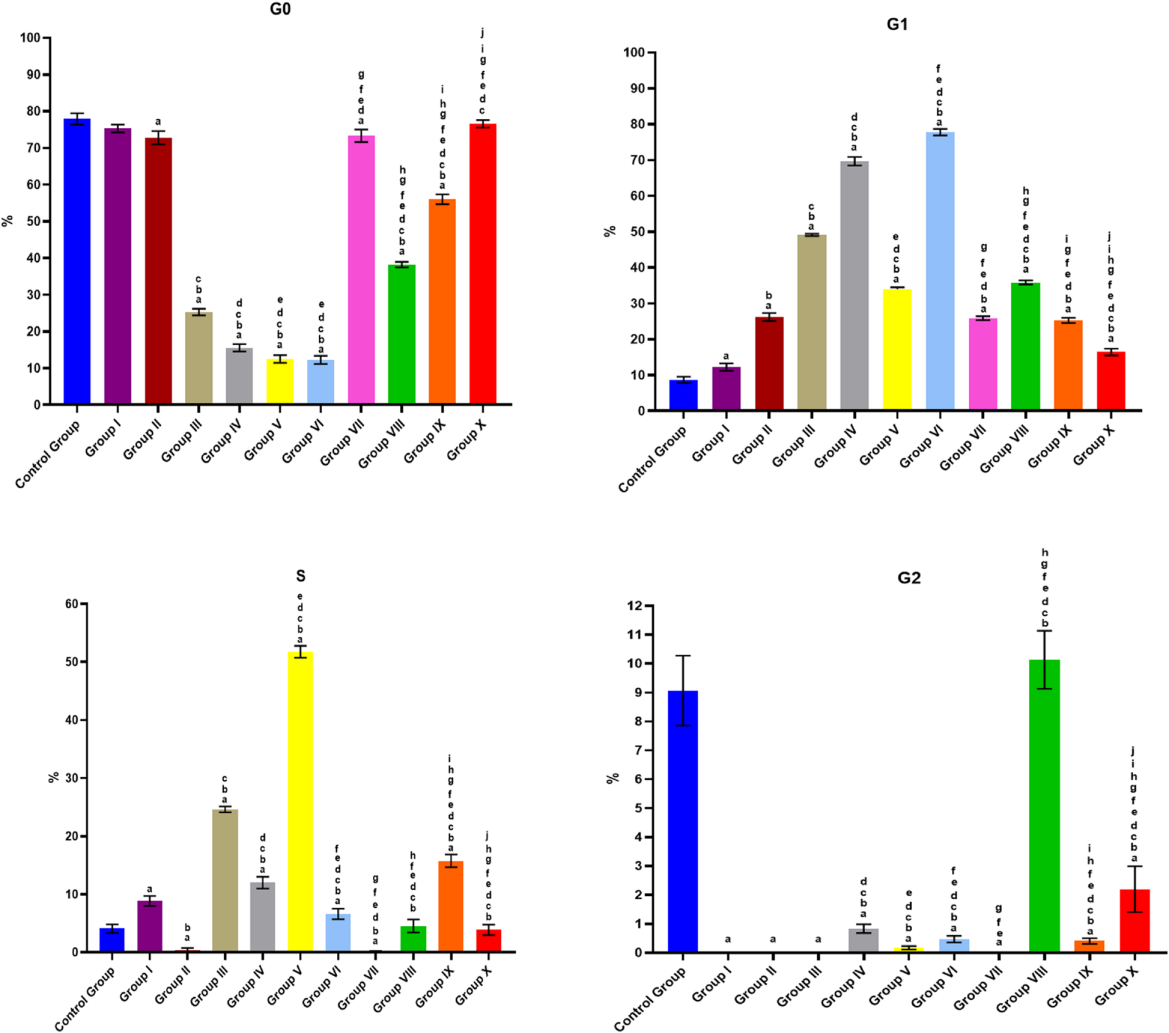
10 (G0, G1, G2, S) for cell cycle analysis

**Fig. 11:**
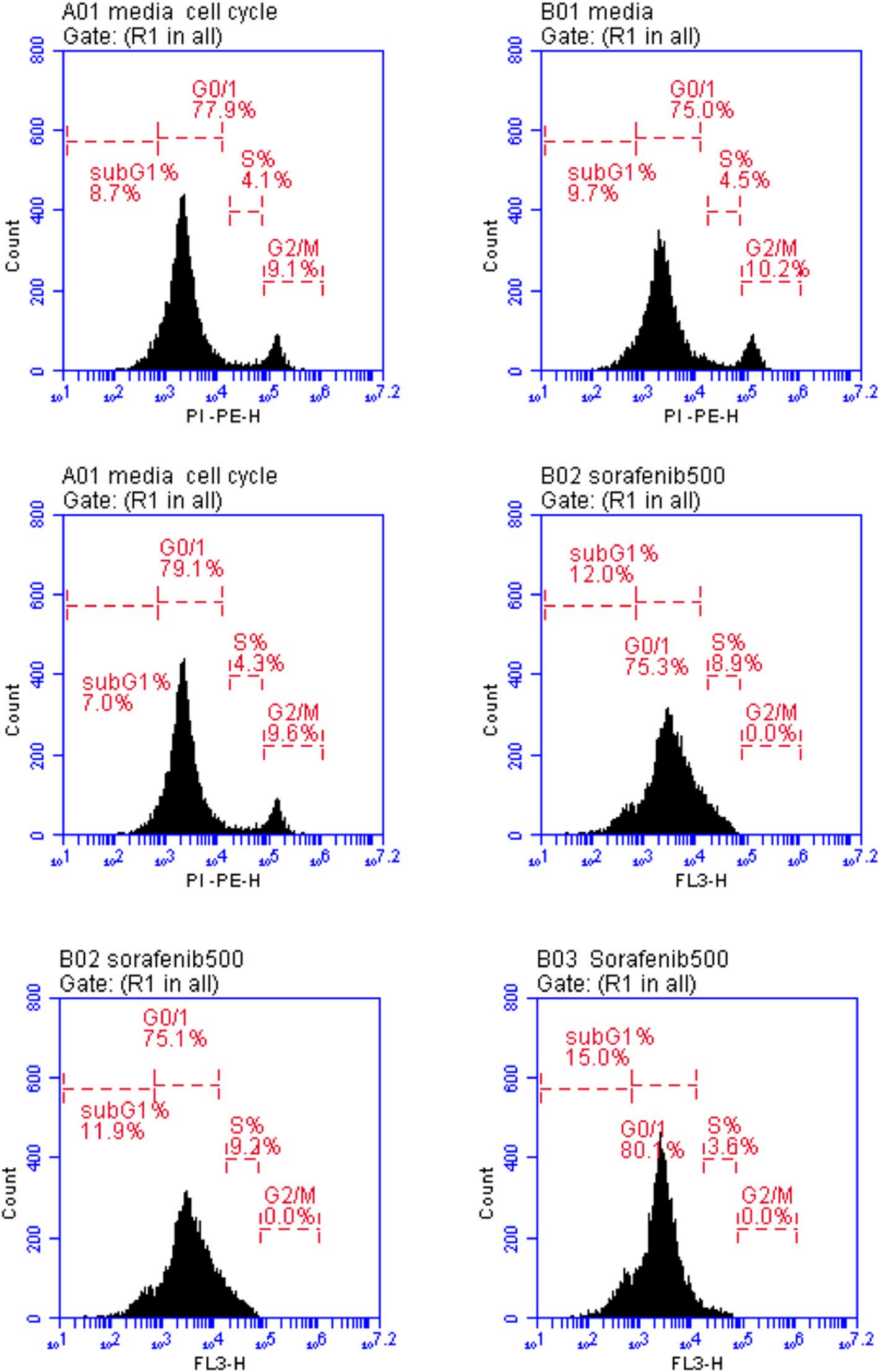
Histogram blot for cell cycle analysis in control and sorafenib groups

**Fig. 12:**
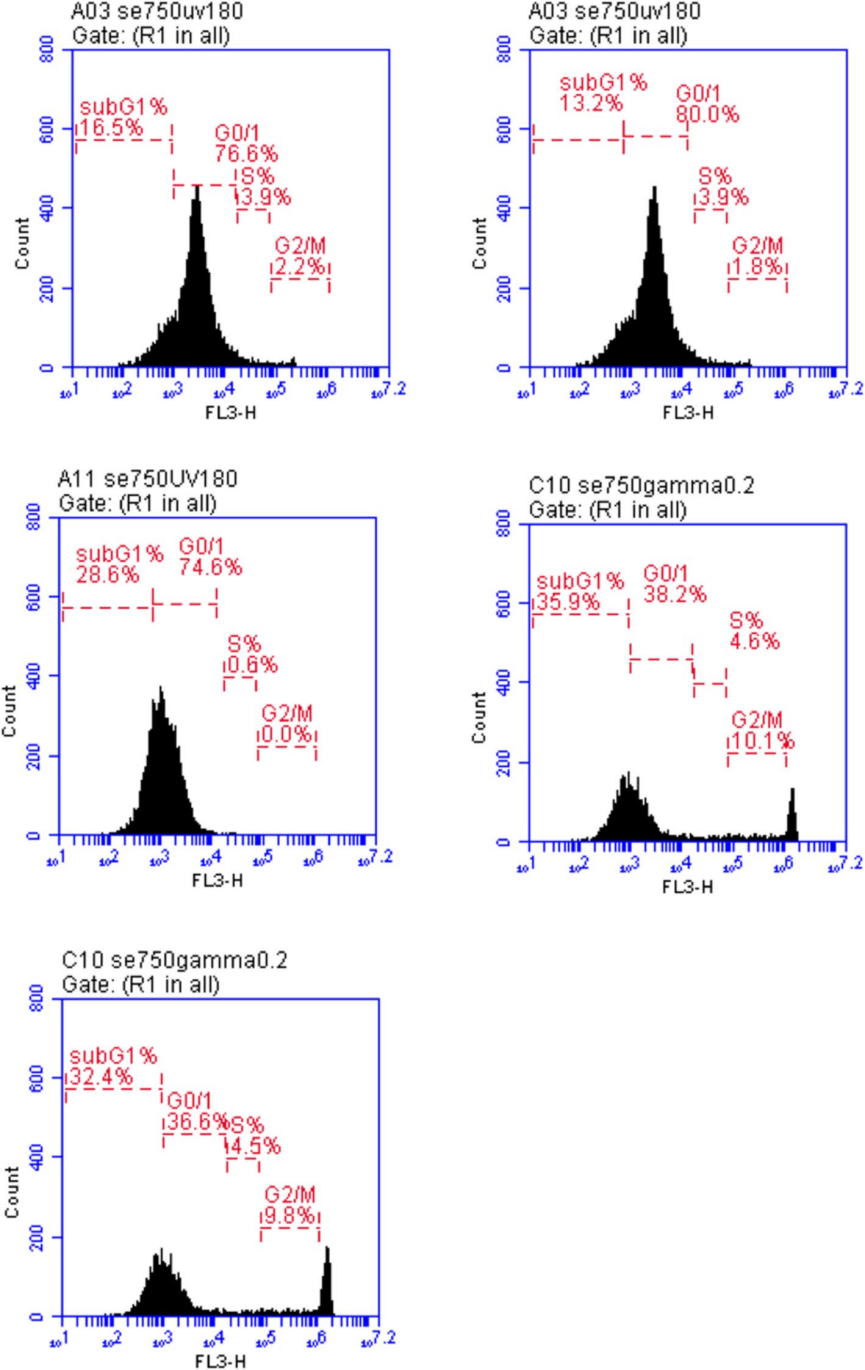
Histogram blot for cell cycle analysis in Se 750 UV180 and Se 750 gamma 0.2 groups

**Fig. 13:**
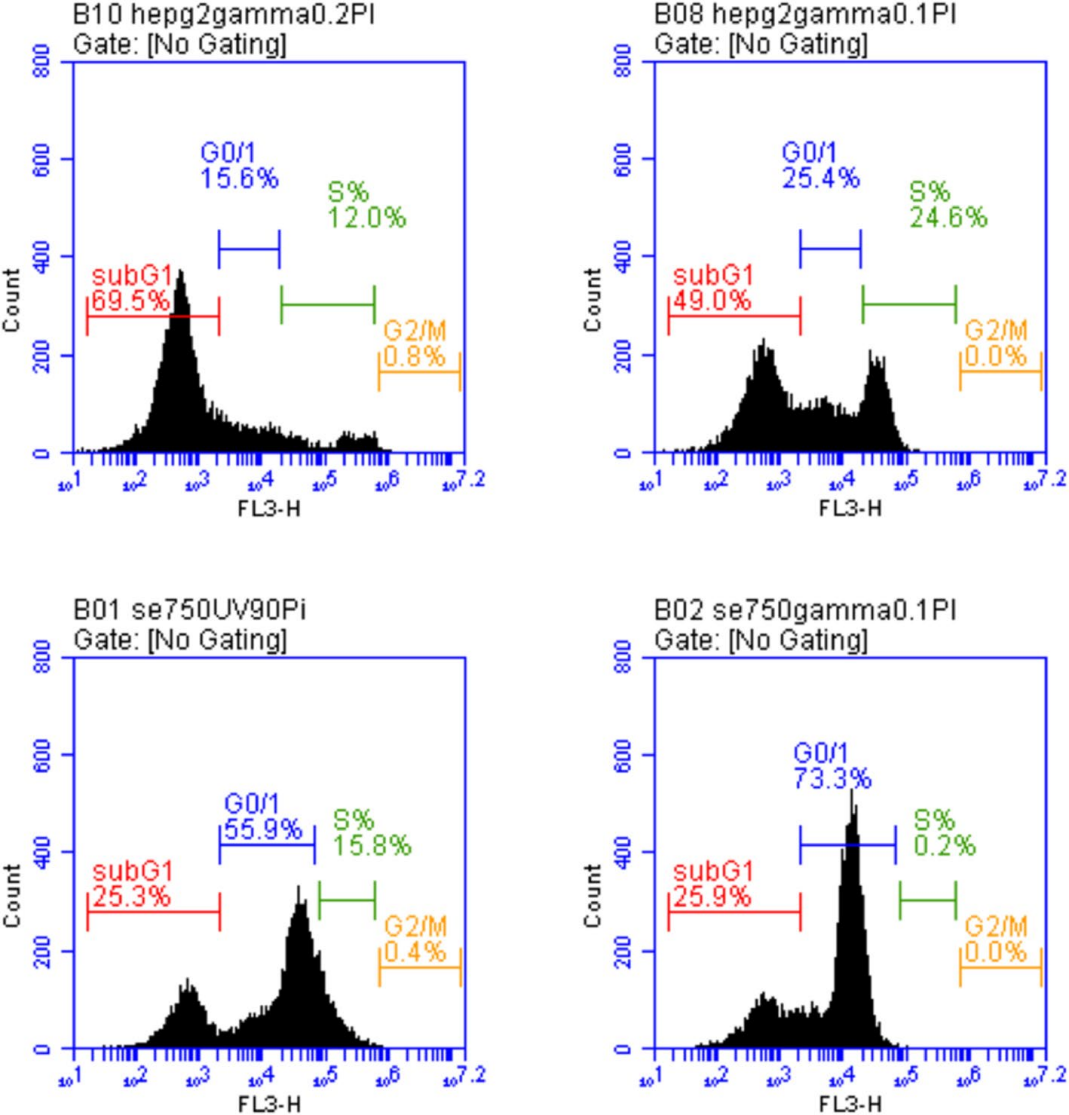
Histogram blot for cell cycle analysis in Se 750 UV90,Hepg2 gamma0.1, Hep g2 gamma 0.2, and Se 750 gamma 0.1 groups

As shown in Fig. 10, according to flowcytometric histogram blots in Figs. 11, 12, and 13 and Table 3, the phases of cell cycle analysis G0 (resting phase), G1 (growth 1 phase), S (DNA synthesis phase), and G2 (rapid growth phase 2). In resting phase, there is an increase in intensity in control group, 1, 2, 10, 7, 9 and decrease in groups 5, 6, 4, and 3. There is no significant difference between sorafenib group and groups 7 and 10. In growth 1 phase, there is a decrease in intensity in control group, 1, 2, 5, 7, 8, 9, and 10 and increase in intensity in groups 6, 4, and 3 (*P*-value (ANOVA) < 0.0001). In S phase, there is a decrease in intensity in group 2, control, 10, 8 and increase in intensity in group 5, 3, 4, and 6. In rapid growth phase, there is an increase in intensity in groups 9 and control group. There is a decrease in intensity in groups 1, 2, 3, 4, 5, 6, 7, 8, and 10 and there is no significant difference between sorafenib group and group 7 (*P*-value (ANOVA) < 0.0001.

**Table 3:** Precise values for cell cycle analysis

Groups	Cell cycle			
	G1(%)	G0 (%)	S (%)	G2 (%)
Control group	8.7 ± 0.9	77.9 ± 1.5	4.1 ± 0.7	9.1 ± 1.2
Group I (Sor)	12.2 ± 1.06^a^	75.3 ± 1.05	8.8 ± 0.87^a^	0^a^
Group II (SeNPs)	26.2 ± 1.1^ab^	72.8 ± 1.8^a^	0.4 ± 0.38^ab^	0^a^
Group III(Gamma 0.1)	49.1 ± 0.35^abc^	25.3 ± 0.9^abc^	24.6 ± 0.5^abc^	0^a^
Group IV(Gamma 0.2)	69.7 ± 1.2^abcd^	15.6 ± 1^abcd^	12 ± 1^abcd^	0.8 ± 0.15^abcd^
Group V(UV 90)	34 ± 0.5^abcde^	12.5 ± 1.1^abcde^	51.8 ± 1^abcde^	0.2 ± 0.1^abcde^
Group VI(UV 180)	77.8 ± 0.92^abcdef^	12.2 ± 1.1^abcde^	6.6 ± 0.92^abcdef^	0.5 ± 0.1^abcdef^
Group VII(SeNPs+Gamma 0.1)	25.9 ± 0.55^abdefg^	73.33 ± 1.7^adefg^	0.2 ± 0.1^abdefg^	0^aefg^
Group VIII(SeNPs+Gamma 0.2)	35.9 ± 0.6^abcdefgh^	38.2 ± 0.76^abcdefgh^	4.5 ± 1.1^bcdefh^	10.13 ± 1^bcdefgh^
Group IX(SeNPs+UV 90)	25.3 ± 0.7^abdefgi^	56 ± 1.4^abcdefghi^	15.8 ± 1.1^abcdefghi^	0.4 ± 0.1^abcdefhi^
Group X(SeNPs+UV 180)	16.4 ± 0.95^abcdefghij^	76.6 ± 1^cdefgij^	3.9 ± 0.9^bcdefghj^	2.2 ± 0.8^abcdefghij^
*P*-value (ANOVA)	< 0.0001	< 0.0001	< 0.0001	< 0.0001

^a^Significant with control group

^b^Significant with DMSO group

^c^Significant with group I

^d^Significant with group II

^e^Significant with group III

^f^Significant with group IV

^g^Significant with group V

^h^Significant with group VI

^i^Significant with group VII

^j^Significant with group VIII

### Cytochrome C by western blotting

#### Cytochrome C in mitochondria

**Fig. 14:**
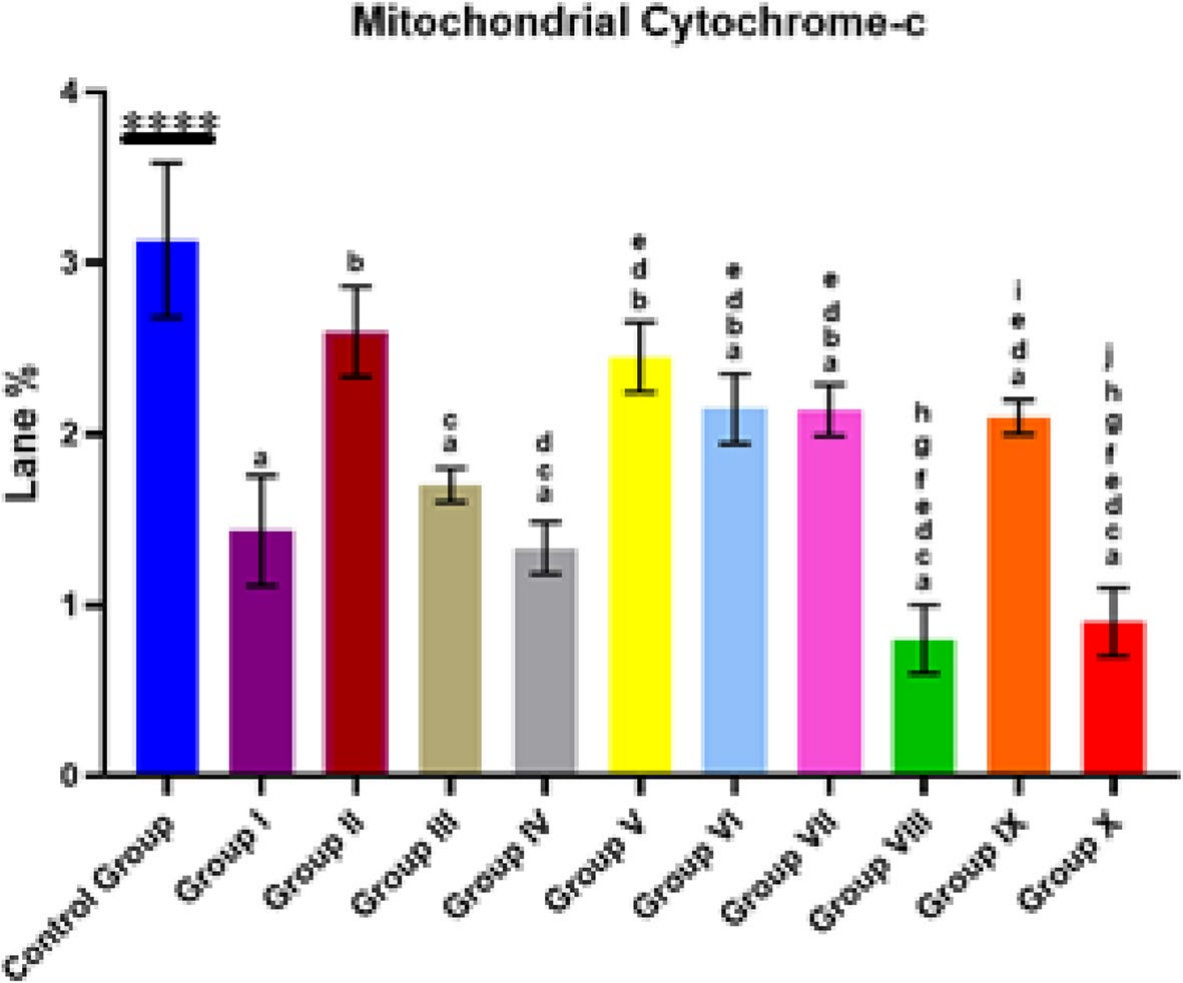
Cytochrome C results in mitochondria

As shown in Fig. 14 control group has the highest concentration in control group, group 10 has the lowest concentration. There is a significant decrease in groups 1, 3, 4, 6, 7, 8, 9, and 10 compared to control group. There is a significant increase in groups 2, 3, 5, 6, and 7, compared to sorafenib group (*P*-value (ANOVA) < 0.0001).

#### Cytochrome C in cytosol

**Fig. 15:**
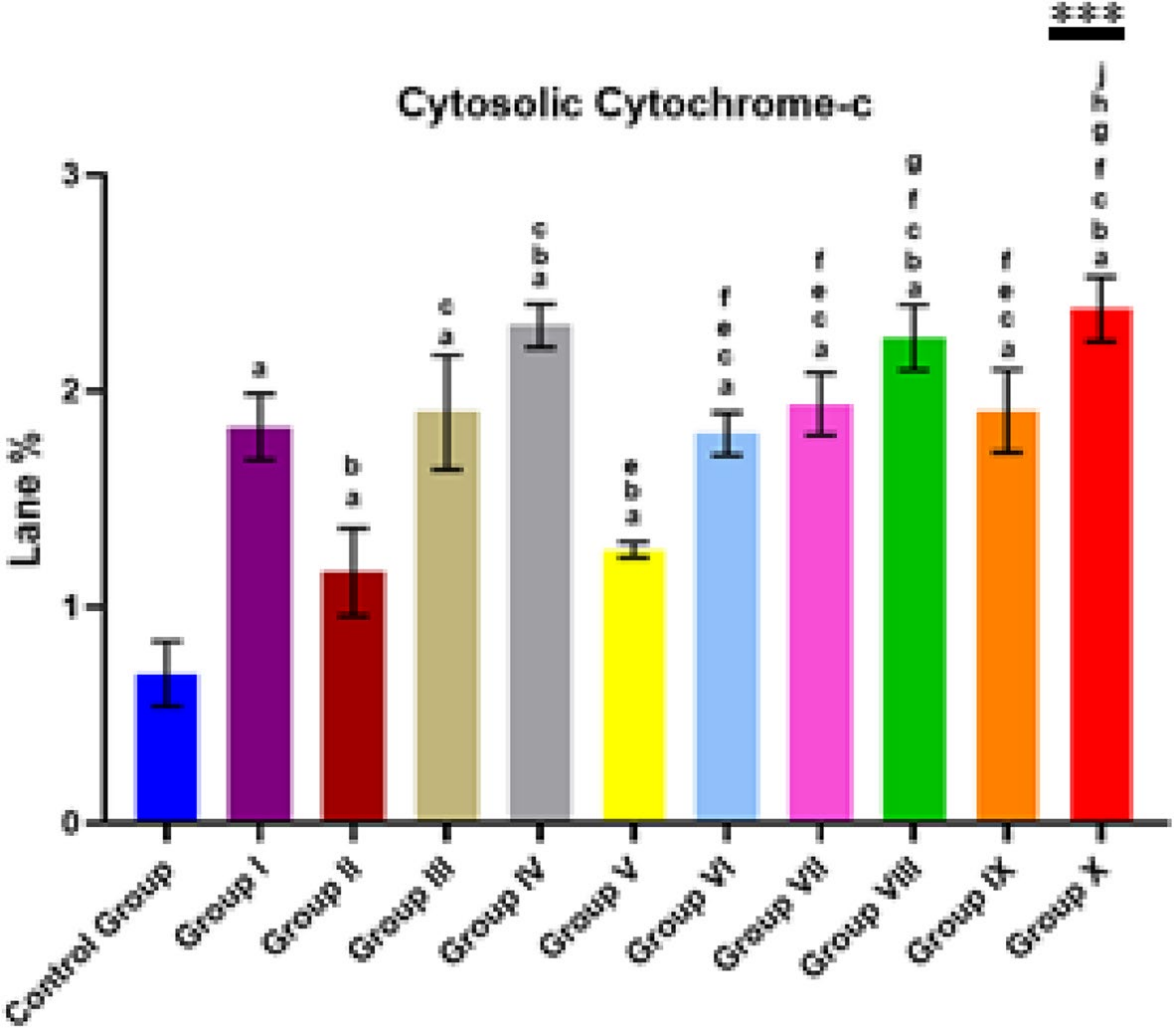
Cytochrome C results in cytoplasm

**Fig. 16:**
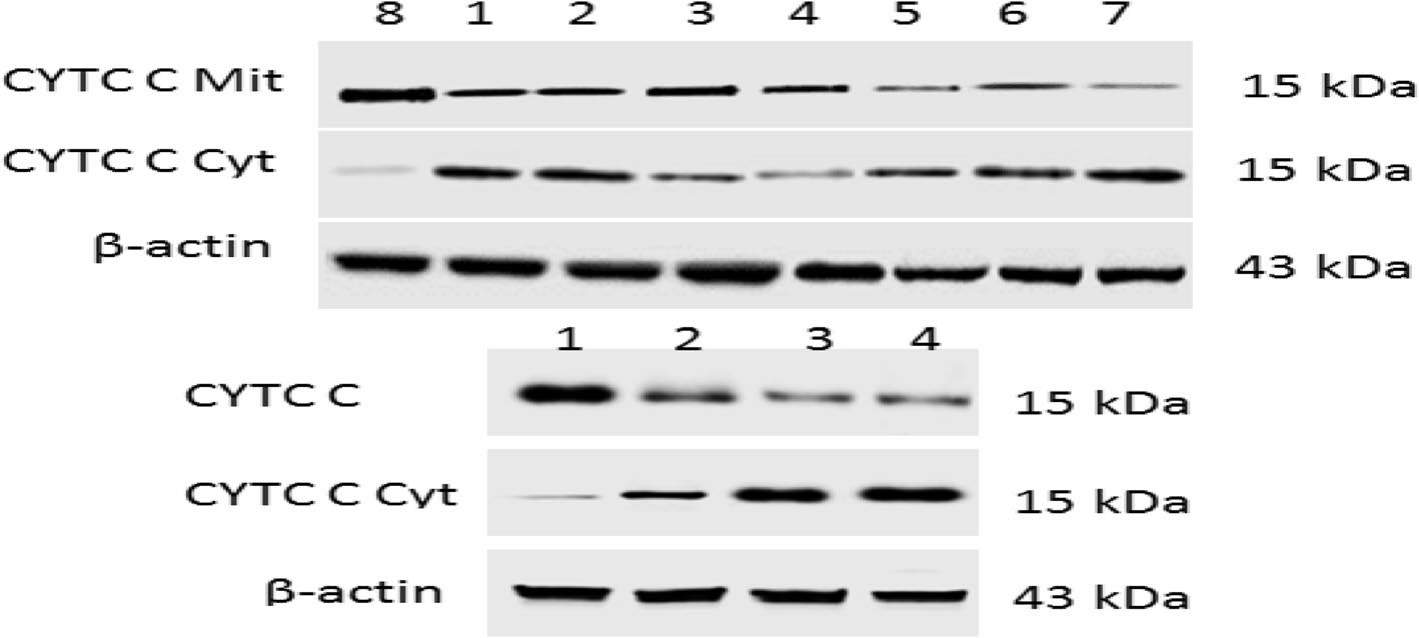
Western blotting bands for Cytochrome C in mitochondria and cytoplasm in experimental groups

As shown in Fig. 15 according to Western blotting bands for Cytochrome C in mitochondria and cytoplasm in Fig. 16, control group has the lowest concentration, group 10 has the highest concentration. There is a significant increase in groups 1, 2, 3, 4, 5, 6, 7, 8, 9, and 10 compared to control group. There is a significant increase in groups 4, 8, and 10 compared to sorafenib group. There is a significant decrease in groups 2 and 5 compared to sorafenib group (*P*-value (ANOVA) < 0.0001).

According to Fig. 15 and Table 4 the untreated HepG2 group (control group) has significantly highest concentration of cytochrome c in mitochondria and the lowest concentration of cytochrome c in cytosole than the other experimental groups. Selenium combined with radiation groups group 10 (Se UV 180) and group 8 (Se gamma 0.2) have the lowest concentration of mitochondrial cytochrome c than untreated HepG2 (control group), Sorafenib group, Selenium only group and radiation only groups.

**Table 4:** Precise values for cytochrome c in cytoplasm and mitochondria

Groups	Mitochondrial Cytochrome-c(Lane %)	Cytosolic Cytochrome-c(Lane %)
Control group	3.1 ± 0.45	0.69 ± 0.15
Group I (Sor)	1.4 ± 0.32^a^	1.83 ± 0.15^a^
Group II (SeNPs)	2.6 ± 0.26^b^	1.16 ± 0.2^ab^
Group III(Gamma 0.1)	1.7 ± 0.1^ac^	1.9 ± 0.26^ac^
Group IV(Gamma 0.2)	1.3 ± 0.15^acd^	2.3 ± 0.1^abc^
Group V(UV 90)	2.45 ± 0.2^bde^	1.26 ± 0.035^abe^
Group VI(UV 180)	2.15 ± 0.2^abde^	1.8 ± 0.1^acef^
Group VII(SeNPs+Gamma 0.1)	2.14 ± 0.15^abde^	1.94 ± 0.14^acef^
Group VIII(SeNPs+Gamma 0.2)	0.8 ± 0.2^acdefgh^	2.24 ± 0.15^abcfg^
Group IX(SeNPs+UV 90)	2.1 ± 0.1^adei^	1.9 ± 0.19^acef^
Group X(SeNPs+UV 180)	0.9 ± 0.2^acdefghj^	2.37 ± 0.15^abcfghj^
*P*-value (ANOVA)	< 0.0001	< 0.0001

^a^Significant with control group

^b^Significant with DMSO group

^c^Significant with group I

^d^Significant with group II

^e^Significant with group III

^f^Significant with group IV

^g^Significant with group V

^h^Significant with group VI

^i^Significant with group VII

^j^Significant with group VIII

### DNA fragmentation

**Fig. 17:**
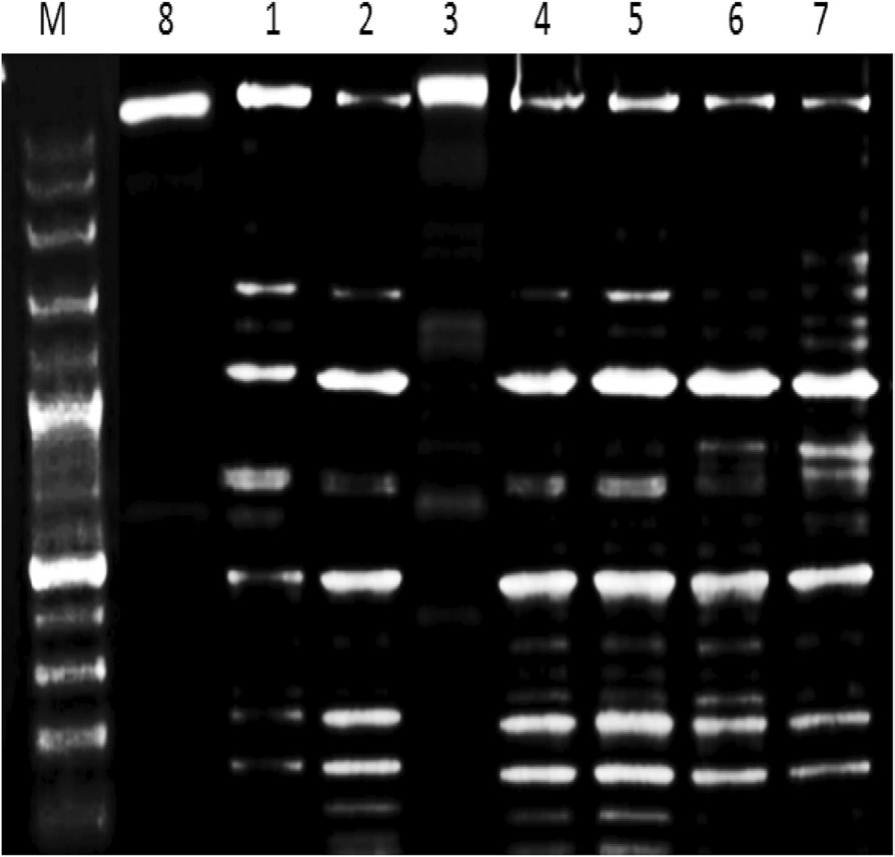
Agarose gel electrophoresis showed DNA fragmentation of all studied groups. Lane M: DNA ladder Lane 8: HepG2 group, Lane 1: Se 750 UV 180, Lane 2: Se 750 γ0.1, Lane 3: Se 750 Lane 4: HepG2 UV 90, Lane 5: HepG2UV 180, Lane 6: HepG2 γ0.1, Lane 7: HepG2 γ0.2

**Fig. 18:**
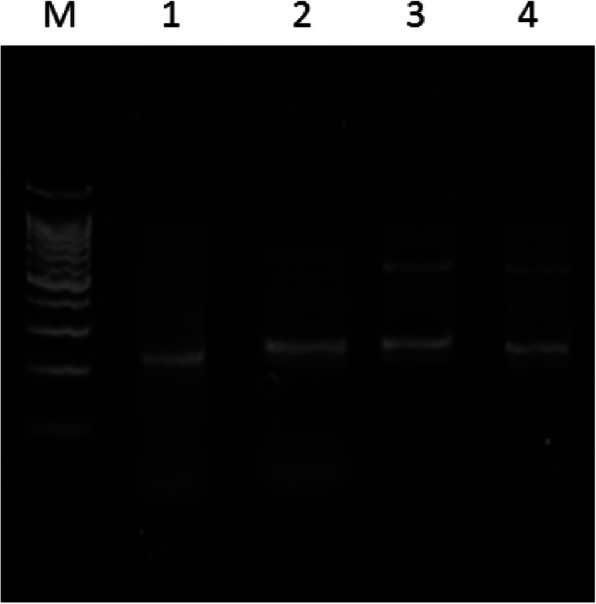
Agarose gel electrophoresis showed DNA fragmentation of all studied groups Lane M: DNA ladder Lane 1: HepG2 group, Lane 2: Sorafenib, Lane 3: Se 750 UV 180, Lane 4: Se 750 γ0.2

As shown in Figs. 17 and 18 the control group did not show DNA fragmentation. Groups of HepG2 UV180, HepG2 UV90, Se 750 γ0.1, Se 750 UV 180, Se750 UV90, and sorafenib showed the highest DNA fragmentation and group Se 750 showed the less fragmentation.

## Discussion

SeNPs’ hepatoprotective, antiviral, and antiparasitic properties have been demonstrated in numerous investigations. In this study, we showed that selenium nanoparticles can directly affect hepatocellular carcinoma cells and trigger their apoptosis, as well as shield the liver from traditional anticancer medications and make their administration easier. However, the hepatoprotective effects of selenium are attributed to the increased production of antioxidant enzymes that contain selenium, and its metabolism results in the generation of reactive oxygen species, which are more prevalent in cancer cells than in normal cells. This characteristic, along with SeNPs’ capacity to cause hepatocellular carcinoma cells to undergo apoptosis, makes them a potentially effective therapeutic treatment, both when used alone and particularly when combined with other anticancer drugs [38, 39].

The Se nanoparticles we characterized had a TEM size of 12.66 nm and a spherical form without aggregation. It has been demonstrated that particles’ diffusion into the cell is facilitated by lowering their size and increasing their surface area [40, 41].

Its particles were neutrally charged since the zeta potential distribution size was 651.8 nm and its potential was − 6.35 mV [42].According to physicochemical principles, particle aggregation occurs when the zeta potential is less than ± 5 mV, while high stability is produced when it is greater than ± 60 mV. There is good stability in the zeta potentials between these two values [43, 44]. Consequently, our synthesized Se NPs exhibit strong cellular uptake and good stability. Additionally, the Se NPs showed a polydispersity index (PDI) of 1.07. Some studies indicate that a limited size distribution is indicated by a PDI value between 0.1 and 0.25, whereas a broad distribution is indicated by a PDI value more than 0.5 [36].

The experimental groups’ percentage of cell viability was lower than that of the control group (Hepg2 media without radiation or selenium). The Sorafenib group’s cell viability was lower than that of the Selenium groups with radiation, selenium groups, and radiation groups, with the exception of group 4 (Hep g2 with gamma radiation 0.2). Selenium mixed with radiation groups, particularly group 4 (gamma 0.2) and group 6 (UV 180), demonstrated greater cell viability than radiation groups lacking selenium in groups 3, 4, 5, and 6. When combined with UV and gamma radiation, selenium nanoparticles reduce the viability of cancer cells while also lessening the strong effects of radiation on cancer cells. This makes selenium nanoparticles an excellent choice for protecting healthy cells from harm in close proximity to cancer cells. Nano-Se can also be used as a radiosensitizer to increase the effectiveness of radiation therapy and lessen side effects than [37]. Combination therapy is better for ongoing local tumor control and better cures than radiation therapy alone, chemotherapy, or sequential therapy [23]. Making Selenium combined with radiation is better than sorafenib drug due to its’ lower side effects. Group 10 (Hep g2 Se UV 180 s) had the lowest cell viability % among selenium and radiation groups, following the findings of our study. According to earlier research, SeNPs can cause cancer cells to undergo apoptosis by activating the p53 and AKT pathways [45, 46], mitochondria-mediated pathways [47, 48], inhibition of the EGFR (epidermal growth factor receptor)-mediated PI3 K/AKT pathway and Ras/Raf/MEK/ERK, activation of MAPK and caspase-3 signaling pathways [23, 47], and these studies showed that p53 can cause cancer cells to undergo apoptosis. P53 is a key player in tumor suppression, mainly through causing growth arrest, apoptosis, and senescence, as well as by preventing angiogenesis [48].

Sequence-specific transcription factors that control the expression of genes that induce apoptosis are encoded by P53. These products, which act directly on mitochondria to induce apoptosis, include Bax (Bcl-2-associated X protein) [49], Noxa (NADPH oxidase activator 1) [50], p53 AIP1 (p53 acetate-induced protein 1) [51], and PUMA (p53-upregulated modulator of apoptosis) [52]. In our study, the concentration of P53 in the control group (Hep G2 only) was significantly lower than that of the other groups, while the highest concentration of P53 was found in group 8 (HepG2 Se gamma 0.2), which was significantly higher than the sorafenib group. Thus, the greatest tumor suppression and apoptosis that took place in group 8 (Se gamma 0.2). The substantial rise in P53 concentration in the Se + radiation groups relative to the selenium group (group 2) supports the superiority of radiation + selenium nanoparticles over selenium nanoparticles alone. Additionally, scientists discovered that SeNPs inhibited tumor growth in animal tests by triggering p53-mediated apoptosis [46]. The mechanism of action of P53 is supported by the data of our study.

The analysis of the cell cycle, which included the G0, G1, S, and G2 phases, revealed that during the resting phase, the cell population increased in the control, sorafenib, selenium, SE UV180, Se gamma 0.1, and Se UV 90 groups and decreased in the UV 90, UV180, gamma 0.2, and gamma 0.1 groups. However, there was no discernible difference between the sorafenib group and the Se gamma 0.1, Se UV180 groups. Phase 1 (growth phase) sees a decline in the number of cells under control, sorafenib, selenium, UV90, Se gamma 0.1, Se gamma 0.2, Se UV 90, and Se UV 180 groups, while the increase in cell population in groups UV 180 and Gamma 0.2 groups occurred.

The cell population in the Selenium, control, Se UV 180, and Se gamma 0.2 groups decreases during the S phase, while the intensity of the UV 90, gamma 0.1, gamma 0.2, and UV 180 groups increases. Groups 9 and the control group have an increase in intensity during the rapid growth phase, while groups Sorafenib, Selenium, Gamma 0.1, Gamma 0.2, UV 90, UV 180, Se gamma 0.1, Se gamma 0.2, and Se UV 180 see a drop in intensity. There is no discernible difference between the groups Se gamma 0.1 and Sorafenib.

According to earlier findings in the G0 phase, untreated HepG2 (control group) and Se coupled with radiation groups exhibit activation, but radiation-only groups exhibit repression Se coupled with radiation groups and the control group are suppressed during the G1 phase, while radiation-only groups are activated. Se coupled with radiation groups and the control group are suppressed during the S phase, while radiation-only groups are activated. The control group (untreated HepG2) and group Se gamma 0.2 are activated during the G2 phase, while the Se group, radiation-only group, and Se combined with radiation group are suppressed. The greatest suppression in Se mixed with radiation groups is found in Group 9 Se UV 90.

When we compared the cell cycle analysis stages of the untreated HepG2 group with those of other groups, particularly in the rapid growth phase G2, we found that the experimental groups had a considerable suppression except group 8 Se gamma 0.2

SeNPs, a novel radiosensitizer, enhanced the killing impact of tumor cells and decreased harm to healthy tissue when used in conjunction with radiation therapy. This, in particular, strengthened radiosensitivity, G2/M phase cell cycle arrest, and autophagy activation [53].

Since the G2 phase is when tumors grow the fastest, treating HCC cancer cells with selenium alone or in combination with radiation inhibits the growth of the tumor [23, 47].

Cytochrome C normally sits in the inner membrane of the mitochondria [54]. The mitochondrial membrane potential (MMP) drops and cytochrome c is released from the mitochondria into the cytoplasm when apoptosis is signaled. Cytochrome c generates apoptosomes in the cytoplasm and activates caspases, including caspase 3, to cause apoptosis [55]. Additionally, studies have demonstrated that intracellular ROS cause the release of cytochrome c into the cytoplasm and decrease MMPs [56, 57].

According to our study, the untreated HepG2 group (Control group) has significantly highest concentration of cytochrome c in mitochondria and the lowest concentration of cytochrome c in cytosole than the other experimental groups. Selenium combined with radiation groups group 10 (Se UV 180) and group 8 (Se gamma 0.2) have the lowest concentration of mitochondrial cytochrome c than untreated HepG2 (control group), Sorafenib group, Selenium only group and radiation only groups. Group 10 (Se UV 180) and group 8 (Se gamma 0.2) showed the highest concentration of cytosolic cytochrome c with a significant increase than untreated HepG2 (control group), Sorafenib, Gamma 0.1, UV 90, UV 180, Selenium gamma 0.1, and Selenium UV 90.

As shown in our study, the concentration of cytosolic cytochrome c in Selenium nanoparticles combined with radiation Group 10 (Se UV 180) and group 8 (Se gamma 0.2) is higher than Sorafenib group which is the commercial drug used for HCC treatment,this indicating the higher apoptotic effect and the higher rate of cancer cells death occurred by Selenium nanoparticles combined with radiation Group 10 (Se UV 180) and group 8 (Se gamma 0.2) and the significant effect of selenium combined with radiation than selenium only [41, 58].

P53 moves into the mitochondria and attaches itself directly to Bcl-XL. The release of cytochrome c from mitochondria to the cytoplasm is induced by p53 mitochondrial translocation and the protein interaction between p53 and Bcl-XL [59]. According to our study there is a correlation between P53 results and cytosolic cytochrome c results in both group 8 (Se gamma 0.2) and group 10 (Se UV 180). The mechanism of action of P53 and cytochrome c is supported by our data.

Cell membrane destruction, cellular contraction, and membrane bleeding are among the chemical and morphological alterations that occur during apoptosis. Caspase activation during biochemical shifts sets off the DNASE enzyme, which damages DNA and manifests as DNA fragmentation [60].

When compared to the untreated Hep G2 group, our investigation revealed that the experimental groups displayed strong evidence of DNA fragmentation, while the untreated Hep G2 group (control group) displayed intact DNA with no symptoms of DNA fragmentation.

Selenium group only showed less DNA fragmentation than experimental groups. Radiation only groups showed more fragmentation than Selenium combined with radiation groups.

According to our study group 10 (Se UV 180) showed more DNA fragmentation in Selenium combined with radiation groups confirming high apoptotic effect in this group than other groups. DNA fragmentation in sorafenib group was significantly low compared to radiation only groups and selenium combined with radiation groups.

Finally by gathering our data, we found that Selenium alone without radiation has a less effect on elimination of cancer cells with high cell viability and less apoptotic effect on the other hand Selenium combined with radiation specially high dose of radiation (UV 180 s and gamma 0.2) has the highest effect on killing cancer cells with significant low cell viability and high apoptotic effect on Hep G2 cell lines, better than radiation only groups. Also, by comparing these data with sorafenib group, we found that sorafenib had a less apoptotic effect than Selenium combined with radiation groups in spite of having the lowest cell viability compared to Selenium combined with radiation groups. So, we can conclude that Selenium combined with radiation UV 180 s and gamma 0.2 have more cell viability than sorafenib group making them lower in side effects than sorafenib drug use and being higher in apoptotic effect than sorafenib group give Selenium combined with radiation (UV 180 s and gamma 0.2) a higher anti cancer effect than sorafenib drug.

## Conclusion

To conclude we found that Selenium alone without radiation has a less effect on elimination of cancer cells with high cell viability and less apoptotic effect on the other hand Selenium combined with radiation specially high dose of radiation UV 180 s and gamma 0.2 has the highest effect on killing cancer cells with significant low cell viability and high apoptotic effect on Hep G2 cell lines, better than radiation only groups. In our study we found also the better effect of Selenium combined with radiation than the current used sorafenib drug by decreasing side effects of selenium combined with radiation compared to sorafenib drug as shown in cell viability results and higher apoptotic effect than sorafenib drug as shown in P53, Cytochrome C and cell cycle analysis results.

Based on the results of this study, we recommend serious application on experimental animals and studying the therapeutic effect of selenium combined with radiotherapy in treating HCC in experimental animals, studying the mortality rate and side effects and comparing them with the use of the drug sorafenib on experimental animals in preparation for future testing on human cases in order to achieve the optimal benefit from the use of selenium combined with radiotherapy as an effective treatment for HCC.

## Data Availability

Data is provided within the supplementary information files.
